# Characterizing brain dynamics during ketamine-induced dissociation and subsequent interactions with propofol using human intracranial neurophysiology

**DOI:** 10.1038/s41467-023-37463-3

**Published:** 2023-03-29

**Authors:** Fangyun Tian, Laura D. Lewis, David W. Zhou, Gustavo A. Balanza, Angelique C. Paulk, Rina Zelmann, Noam Peled, Daniel Soper, Laura A. Santa Cruz Mercado, Robert A. Peterfreund, Linda S. Aglio, Emad N. Eskandar, G. Rees Cosgrove, Ziv M. Williams, R. Mark Richardson, Emery N. Brown, Oluwaseun Akeju, Sydney S. Cash, Patrick L. Purdon

**Affiliations:** 1 Department of Anesthesia, Critical Care and Pain Medicine, Massachusetts General Hospital, Harvard Medical School, Boston, MA USA; 2 Department of Biomedical Engineering, Boston University, Boston, MA USA; 3 Department of Radiology, MGH/HST Martinos Center for Biomedical Imaging and Harvard Medical School, Boston, MA USA; 4 Institute for Medical Engineering and Sciences, Department of Electrical Engineering and Computer Science, Research Laboratory of Electronics, Massachusetts Institute of Technology, Cambridge, MA USA; 5 Department of Brain and Cognitive Sciences, Massachusetts Institute of Technology, Cambridge, MA USA; 6 Picower Institute for Learning and Memory, Massachusetts Institute of Technology, Cambridge, MA USA; 7 Department of Neurology, Massachusetts General Hospital, Harvard Medical School, Boston, MA USA; 8 Center for Neurotechnology and Neurorecovery, Massachusetts General Hospital, Boston, MA USA; 9 Department of Anesthesiology, Perioperative and Pain Medicine, Brigham and Women’s Hospital, Boston, MA USA; 10 Department of Neurological Surgery, Albert Einstein College of Medicine, Bronx, NY USA; 11 Department of Neurosurgery, Brigham and Women’s Hospital, Boston, MA USA; 12 Department of Neurosurgery, Massachusetts General Hospital, Harvard Medical School, Boston, MA USA

**Keywords:** Neural circuits, Neurophysiology

## Abstract

Ketamine produces antidepressant effects in patients with treatment-resistant depression, but its usefulness is limited by its psychotropic side effects. Ketamine is thought to act via NMDA receptors and HCN1 channels to produce brain oscillations that are related to these effects. Using human intracranial recordings, we found that ketamine produces gamma oscillations in prefrontal cortex and hippocampus, structures previously implicated in ketamine’s antidepressant effects, and a 3 Hz oscillation in posteromedial cortex, previously proposed as a mechanism for its dissociative effects. We analyzed oscillatory changes after subsequent propofol administration, whose GABAergic activity antagonizes ketamine’s NMDA-mediated disinhibition, alongside a shared HCN1 inhibitory effect, to identify dynamics attributable to NMDA-mediated disinhibition versus HCN1 inhibition. Our results suggest that ketamine engages different neural circuits in distinct frequency-dependent patterns of activity to produce its antidepressant and dissociative sensory effects. These insights may help guide the development of brain dynamic biomarkers and novel therapeutics for depression.

## Abstract (web summary)

The neural mechanisms underpinning ketamine’s dissociative and antidepressant effects remain poorly understood. Here, the authors analyzed ketamine-induced brain dynamics with intracranial recordings in humans and found that ketamine engages different brain areas in distinct frequency-dependent patterns that may relate to its dissociative and antidepressant effects.

## Introduction

Ketamine is a dissociative anesthetic that has both anesthetic and psychoactive properties^1,2^. Intravenous induction doses (1–2 mg/kg) of ketamine result in a rapid loss of consciousness appropriate for general anesthesia^3,4^. At subanesthetic doses (0.5 mg/kg), ketamine produces a dissociative state, which includes gaps in memory, out-of-body experiences, and altered sensory perception^5–7^. In addition, intravenous administration of a subanesthetic dose of ketamine induces significant and rapid antidepressant-like response in depressed patients^8^. Although ketamine was approved by the Food and Drug Administration (FDA) for adult patients with treatment-resistant depression^9^, the neuropsychiatric side effects have limited its extensive use in clinical practice^10,11^. Defining the neural circuits engaged in ketamine’s rapid antidepressant and dissociative effects is an important priority that could facilitate the development of improved therapies with fewer side effects and greater safety.

Ketamine is known to induce profound changes in brain oscillatory dynamics that appear to be correlated with its antidepressant and sensory dissociative activity^7,12–18^. The electrophysiologic profile of subanesthetic ketamine in humans generally includes an increase of gamma oscillation power and a decrease of delta, alpha, and beta oscillation power^7,12–14^. Oscillatory power changes have also been reported in patients with depression and have been used to differentiate depressive from healthy subjects^15^. However, the relationships between these changes in oscillatory power and the neural circuit mechanisms of depression and dissociation are not well-understood. Previous studies suggest that at subanesthetic doses, ketamine preferentially blocks the NMDA receptors on GABAergic inhibitory interneurons, resulting in the disinhibition of downstream excitatory pyramidal neurons that is thought to facilitate increased gamma-band activity^19–21^. When GABA_A_ agonists, such as benzodiazepines, are administered alongside ketamine, they mitigate dissociations, possibly by restoring inhibitory activity in the affected brain regions^22,23^. In addition, ketamine inhibits the hyperpolarization-activated cyclic nucleotide-gated potassium channel 1 (HCN1), a molecular target that is thought to play an important role in generating rhythmic EEG activity and is considered a novel therapeutic target for depressive disorders^24–28^. Studies have been conducted to investigate which cortical or subcortical structures play a major role in mediating this process. Previous work has showed that ketamine’s antidepressant effects are largely dependent upon its actions within the prefrontal cortex and the hippocampus^29^. On the other hand, the reduction of alpha oscillations in the precuneus and temporal-parietal junction and the 3 Hz rhythm in the deep posteromedial cortex (PMC), as studied in rodents and a human patient, have been proposed as mechanisms for ketamine-induced dissociation^7,30,31^. Functional connectivity analysis with fMRI and EEG suggest that ketamine disrupts the frontoparietal default mode network connectivity^12,32^. Although ketamine’s antidepressive and dissociative effects are known to co-occur whenever the drug is administered, these effects may in fact be mediated by distinct mechanisms within distinct neural circuits. If that were true, it might be possible to design novel therapeutics with greater specificity and fewer side effects.

In this study, we measured intracranial EEG (iEEG) in human patients implanted with intracranial electrodes who were administered a subanesthetic dose of ketamine prior to induction of general anesthesia with propofol for electrode removal surgery. Our goal was to characterize the brain regions involved in different ketamine-induced rhythms in order to better understand their potential role in mediating ketamine’s dissociative and antidepressant properties. In addition to characterizing changes in canonical frequency bands associated with subanesthetic ketamine, we also looked for evidence of a 3 Hz rhythm recently implicated in ketamine-induced dissociation^30^. To characterize the potential role of NMDA and HCN1 receptors in producing ketamine-induced oscillations, we analyzed the interactions between subanesthetic ketamine and propofol. Propofol is a positive GABA allosteric modulator and HCN1 blocker^33,34^. Propofol’s GABAergic activity would be expected to antagonize any ketamine-induced oscillations stemming from NMDA-mediated disinhibition. At the same time, propofol would be expected to further potentiate any ketamine-induced oscillations originating from HCN1 inhibition.

## Results

We collected data from 10 epilepsy patients implanted with intracranial depth electrodes to identify sites of epileptogenic origin (Table 1, Supplementary Fig. 1 and Supplementary Video). The responses on the abbreviated Clinician-Administered Dissociative States Scale (CADSS)^35–37^ questionnaire (Supplementary Fig. 2) are summarized in Supplementary Table 1. The responses on the questionnaire are consistent with a dissociative state induced by subanesthetic ketamine.

**Table 1:** Subject demographic and clinical information

Subject ID	Gender	Age	Depth Electrodes	Bipolar Channels	Baseline Duration (s)	Ketamine Duration (s)	Propofol Duration (s)	Drugs Given During General Anesthesia Period
1	F	59	10	55	366	840	NA^a^	Propofol, fentanyl, midazolam
2	M	22	11	57	370	739	164	Propofol, remifentanil, rocuronium
3	F	28	12	68	341	705	235	Propofol, fentanyl, rocuronium
4	M	22	10	60	307	850	NA^a^	Propofol
5	M	48	14	77	201	840	57	Propofol
6	F	34	9	65	300	840	402	Propofol (200 mg), fentanyl (100 mcg), rocuronium (30 mg), succinylcholine (100 mg)
7	F	22	10	104	300	840	50	Propofol (200 mg), fentanyl (100 mcg), midazolam (2 mg), rocuronium (40 mg), lidocaine (60 mg), vancomycin (1 g)
8	M	48	9	102	300	840	NA^a^	Propofol (200 mg), fentanyl (100 mcg), rocuronium (50 mg), lidocaine (80 mg), vancomycin (1 g), clindamycin (900 mg)
9	M	33	11	121	300	840	235	Propofol (200 mg), fentanyl (150 mcg), rocuronium (50 mg), lidocaine (80 mg), vancomycin (1250 mg)
10	F	43	10	115	300	840	445	Propofol (100 mg), rocuronium (50 mg)

^a^We did not collect the data for this patient because of limited time in the operating room. Dose information is not available for subjects #1-5. *F* female, *M* male, *NA* not available.

### Ketamine and propofol-induced location- and frequency-dependent iEEG dynamics

**Fig. 1:**
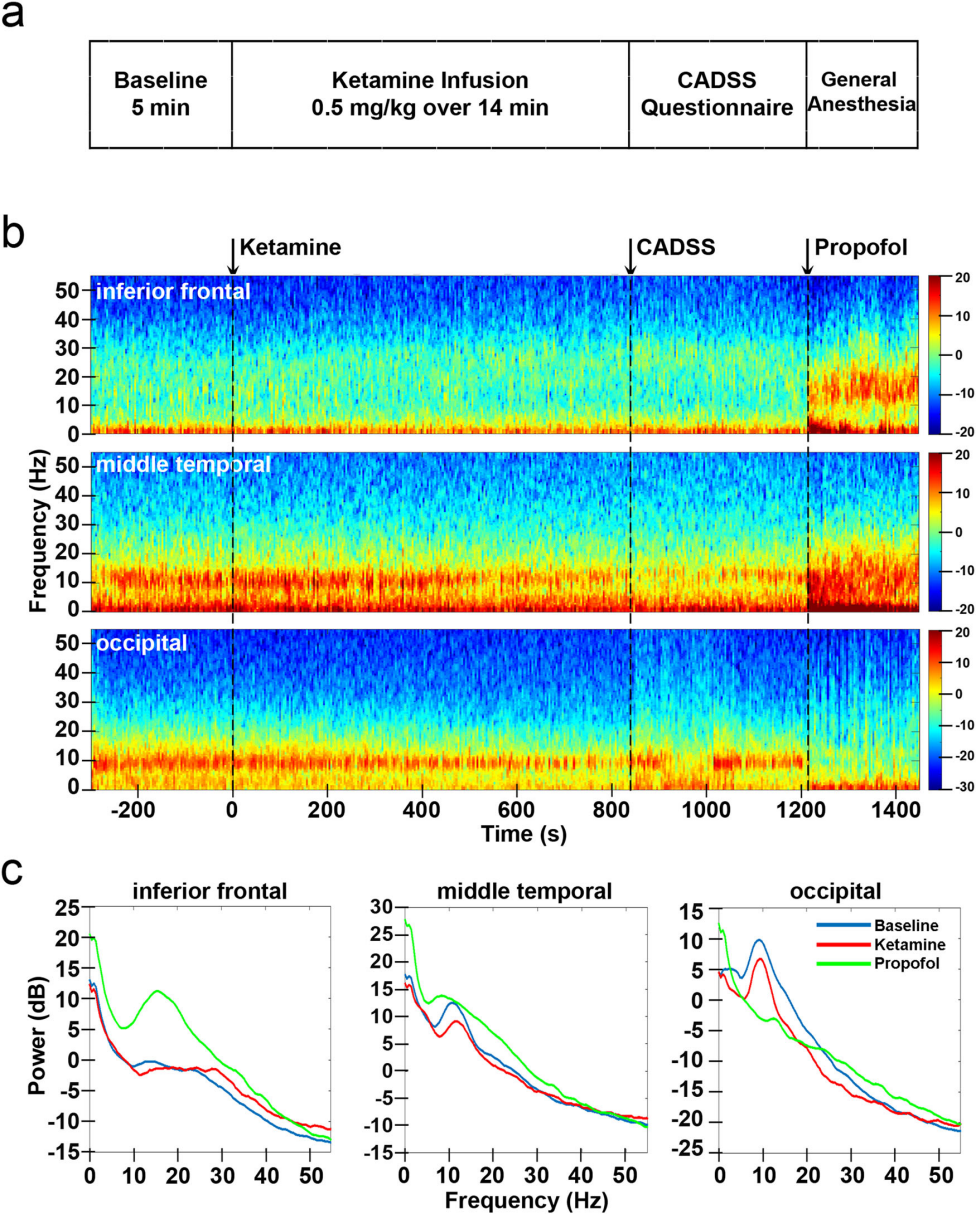
Study protocol and intracranial EEG power changes for example channels. **a** Study protocol. **b** Power spectrogram (dB) for three example channels: inferior frontal, middle temporal and occipital from Subject #9. **c** Power spectrum averaged across time during baseline, ketamine, and propofol conditions for the three example channels. CADSS: clinician-administered dissociative states scale. Source data are provided as a Source Data file Fig. 1.

We observed distinct dynamic patterns in the iEEG after ketamine infusion, which changed after the administration of propofol. Figure 1 shows the spectrogram and power spectra for 3 channels in the inferior frontal, middle temporal, and occipital cortices from an example subject. The spectrograms for other subjects are shown in Supplementary Fig. 3. Under ketamine, we observed increased gamma power (25–55 Hz) in the inferior frontal channel and decreased alpha power (8–15 Hz) in the middle temporal and occipital channels. After propofol was added, there was a large increase of power in the inferior frontal and middle temporal channels for nearly all frequencies, except for upper gamma band (40–55 Hz). In contrast, the reduction of alpha oscillations in the occipital channels was further enhanced with the addition of propofol. These results suggest that the iEEG dynamics induced by ketamine and propofol are location- and frequency-dependent. To understand how these brain dynamics mapped to different brain structures, we analyzed the changes in power for different cortical and subcortical structures, first after ketamine infusion and then after the addition of propofol.

### Ketamine induced an increase in gamma oscillation power and a reduction of low-frequency oscillation power

**Fig. 2:**
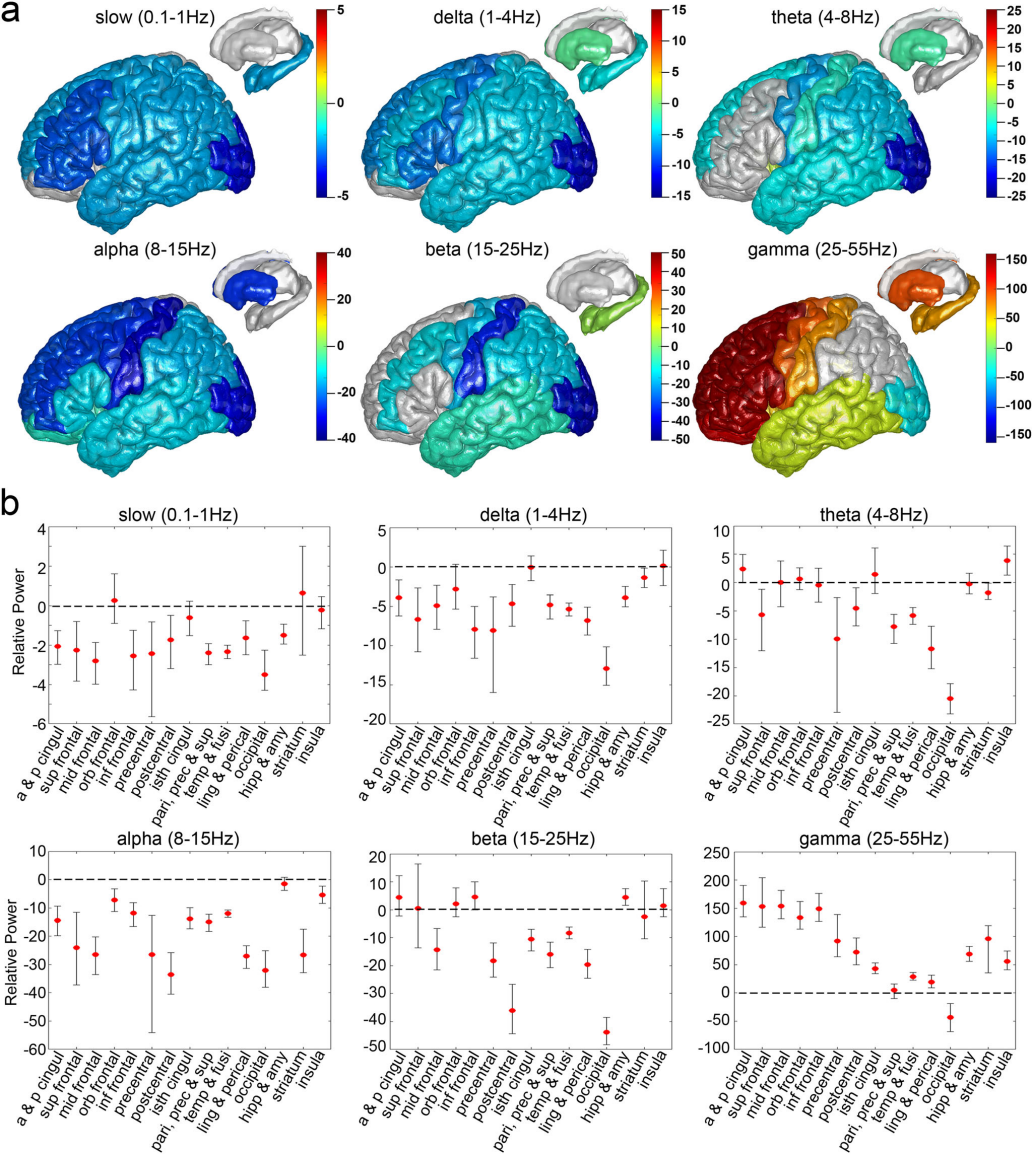
Structural mapping for intracranial EEG power changes after ketamine infusion relative to baseline at 6 frequencies. **a** The mean differences in power (dB) after ketamine infusion relative to baseline were calculated for 824 channels across 10 subjects and plotted on Colin 27 brain template. Warmer colors indicate an increase in power, cooler colors indicate a decrease in power, and a grey color indicates no change in power (confidence interval overlaps zero). **b** Mean and bootstrap 95% confidence interval for intracranial EEG power changes after ketamine infusion relative to baseline at 6 frequencies for 15 structural labels: a & p cingul (anterior and posterior cingulate), sup frontal (superior frontal), mid frontal (middle frontal), orb frontal (orbitofrontal), inf frontal (parsopercularis, parsorbitalis, and parstriangularis), precentral, postcentral, isth cingul (isthmuscingulate), pari, prec & sup (parietal, precuneus, and supramarginal), temp & fusi (temporal and fusiform), ling & perical (lingual and pericalcarine), occipital, hipp & amy (hippocampal and amygdala), striatum (caudate and putamen), and insula. See Supplementary Table 2 for a detailed description of channel and subject numbers by brain region. Source data are provided as a Source Data file Fig. 2.

We analyzed the changes in iEEG dynamics for different brain structures after ketamine infusion (Fig. 2, Supplementary Table 2 and Supplementary Fig. 4). For gamma frequencies (25-55 Hz), a greater than 100 dB increase in mean power after ketamine infusion compared with baseline was detected in frontal structures, which include the anterior and posterior cingulate (159.04 dB), superior frontal (153.03 dB), middle frontal (153.59 dB), orbitofrontal (133.68 dB), and inferior frontal (149.20 dB) areas. The mean power increase in precentral, postcentral, isthmus cingulate, temporal structures, lingual, pericalcarine, hippocampus, amygdala, striatum, and insula, was between 19.07 and 96.37 dB. A decrease in mean gamma power was detected in occipital channels (−42.96 dB). For beta frequencies (15-25 Hz), while an increase in power was detected in hippocampus and amygdala (4.43 dB), a decrease in power was detected for middle frontal (−14.26 dB), precentral (−18.21 dB), postcentral (−36.00 dB), isthmus cingulate (−10.53 dB), parietal (−15.90 dB) and temporal structures (−8.40 dB), as well as the lingual and pericalcarine (−19.58 dB), and the occipital cortices (−43.81 dB). No other structural labels showed changes in power after ketamine infusion (i.e., confidence intervals overlapped zero). For alpha frequencies (8-15 Hz), the decrease of mean alpha power was observed for nearly all structure labels with the largest reduction in postcentral (−33.55 dB) and occipital cortices (−32.07 dB). For theta rhythms (4-8 Hz), we identified an increase of power in insula (3.88 dB) cortex and decrease of power in superior frontal (−5.65 dB), precentral (−9.96 dB), postcentral (−4.50 dB), parietal (−7.75 dB) and temporal structures (−5.83 dB), lingual and pericalcarine (−11.68 dB), as well as the occipital cortices (−20.52 dB) and striatum (−1.85 dB). For slow (0.1-1 Hz) and delta frequencies (1-4 Hz), the decrease in power was observed in most of the structural labels (slow: −1.51 to −3.51 dB, delta: −1.40 to −12.91 dB), except for orbitofrontal, isthmus cingulate, striatum, and insula cortex, which did not showed changes in power after ketamine infusion.

### Propofol reversed the gamma band iEEG dynamics induced by ketamine in frontal regions and caused a further reduction of occipital alpha oscillation power

**Fig. 3:**
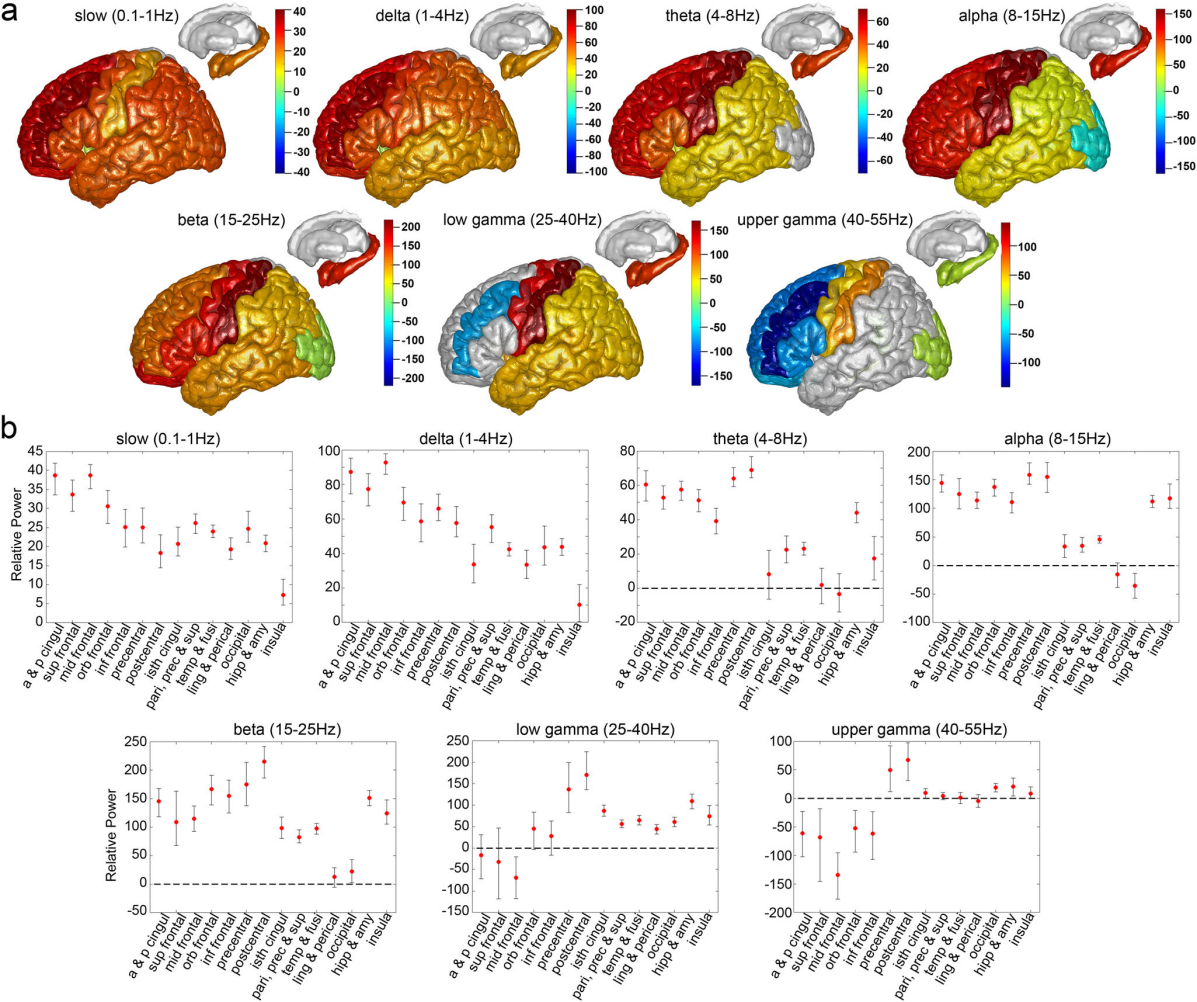
Structural mapping for intracranial EEG power changes after propofol bolus relative to ketamine period at 7 frequencies. **a**, The mean differences in power (dB) after propofol bolus relative to ketamine period were calculated for 606 channels across 7 subjects and plotted on Colin27 brain template. Warmer colors indicate an increase in power, cooler colors indicate a decrease in power, and a grey color indicates no change in power (confidence interval overlaps zero). **b**, Mean and bootstrap 95% confidence interval for intracranial EEG power changes after propofol bolus relative to ketamine period at 7 frequencies for 14 structural labels: a & p cingul (anterior and posterior cingulate), sup frontal (superior frontal), mid frontal (middle frontal), orb frontal (orbitofrontal), inf frontal (parsopercularis, parsorbitalis, and parstriangularis), precentral, postcentral, isth cingul (isthmuscingulate), pari, prec & sup (parietal, precuneus, and supramarginal), temp & fusi (temporal and fusiform), ling & perical (lingual and pericalcarine), occipital, hipp & amy (hippocampal and amygdala), and insula. See Supplementary Table 3 for a detailed description of channel and subject numbers by brain region. Source data are provided as a Source Data file Fig. 3.

Adding the propofol (Fig. 3, Supplementary Table 3 and Supplementary Fig. 5) reversed the gamma power (40–55 Hz) increase in anterior and posterior cingulate (−61.26 dB), superior frontal (−68.32 dB), middle frontal (−134.51 dB), orbitofrontal (−52.27 dB), and inferior frontal (−61.86 dB) regions of the brain, as well as the gamma power decrease in the occipital cortex (18.85 dB). In addition, propofol further intensified the gamma power increase at precentral (49.39 dB), postcentral (67.34 dB), isthmus cingulate (9.78 dB), hippocampus and amygdala (20.56 dB). The presence of propofol reversed the alpha power (8-15 Hz) decrease induced by ketamine for most of the structural labels (33.33 to 158.32 dB) except for occipital cortices (−35.20 dB), which showed a further reduction in power after propofol administration. In addition, propofol increased the beta power (15-25 Hz) for nearly all structural labels (22.00 to 214.90 dB). For theta rhythms (4-8 Hz), propofol also increased theta power in most of the structural labels (17.35 to 68.88 dB). The addition of propofol reversed the power reduction induced by ketamine at slow (0.1-1 Hz, 7.31 to 38.66 dB) and delta (1-4 Hz, 10.29 to 92.64 dB) oscillations for all the structural labels.

### Subanesthetic doses of ketamine induced an increase of 3 Hz oscillation in posteromedial cortex (PMC)

**Fig. 4:**
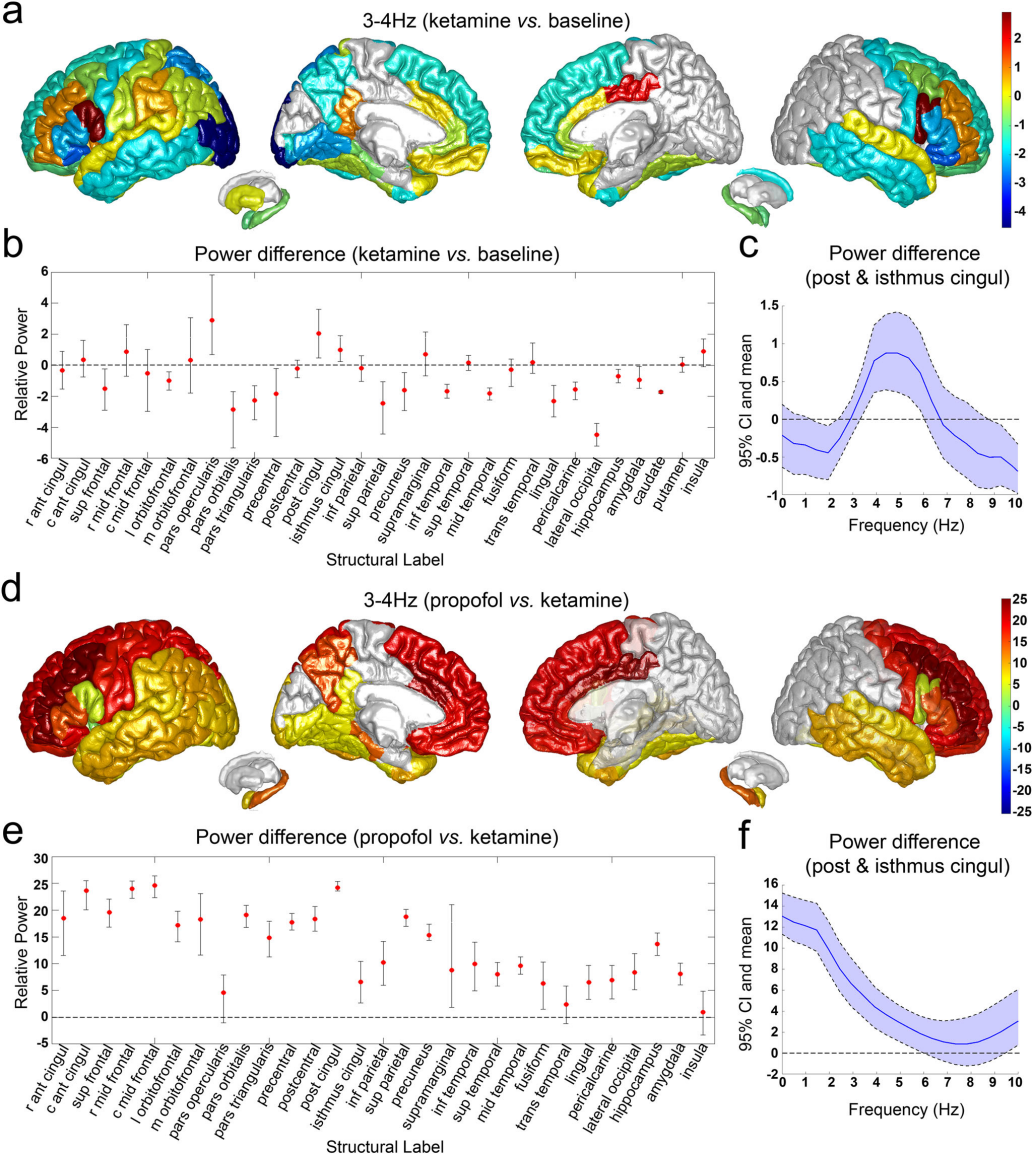
Analysis of 3–4 Hz intracranial EEG power changes after administration of ketamine and propofol. **a**, Structural mapping for 3–4 Hz intracranial EEG power (dB) changes after ketamine infusion. **b**, Mean and bootstrap 95% confidence interval (CI) for 3–4 Hz intracranial EEG power (dB) changes after ketamine infusion. **c** Power spectrum for posterior and isthmus cingulate cortex after ketamine infusion. Data are presented as mean and bootstrap 95% CI. **d** Structural mapping for 3–4 Hz intracranial EEG power (dB) changes after propofol bolus. **e** Mean and bootstrap 95% CI for 3–4 Hz intracranial EEG power (dB) changes after propofol bolus. **f** Power spectrum for posterior and isthmus cingulate cortex after propofol bolus. Data are presented as mean and bootstrap 95% CI. r ant cingul: rostral anterior cingulate; c ant cingul: caudal anterior cingulate; sup frontal: superior frontal; r mid frontal: rostral middle frontal; c mid frontal: caudal middle frontal; l orbitofrontal: lateral orbitofrontal; m orbitofrontal: medial orbitofrontal; post cingul: posterior cingulate; isthmus cingul: isthmus cingulate; inf parietal: inferior parietal; inf temporal: inferior temporal; sup temporal: superior temporal; mid temporal: middle temporal; trans temporal: transverse temporal. See Supplementary Table 4 for a detailed description of channel and subject numbers by brain region. Source data are provided as a Source Data file Fig. 4.

We studied the spatial distribution of 3 Hz rhythms after the administration of ketamine and propofol (Fig. 4, Supplementary Table 4 and Supplementary Fig. 6). We identified a dramatic increase of 3-4 Hz oscillatory power after ketamine infusion in posterior (2.05 dB) and isthmus (1.00 dB) cingulate cortex, which are part of the PMC, as well as the pars opercularis (2.90 dB) located within the inferior frontal cortex (Fig. 4a, b). We then analyzed the spectrum of the oscillatory activity within PMC by plotting the power differences after ketamine relative to baseline for posterior and isthmus cingulate cortex as a function of the frequency (Fig. 4c). We found that the increase of iEEG power after ketamine peaked between 3 to 6 Hz. The addition of propofol greatly increased the 3-4 Hz power in most brain regions (6.34 to 24.57 dB), including the posterior and isthmus cingulate cortex, suggesting that the effects of ketamine and propofol on this 3-4 Hz rhythm may be additive rather than antagonistic (Fig. 4d, e).

## Discussion

In this study, we show, in humans, a detailed description of the principal oscillatory changes in cortical and subcortical structures after the administration of a subanesthetic dose of ketamine. Using intraoperative recordings from intracranial electrodes in 10 patients with epilepsy, we found that ketamine increased gamma oscillations within prefrontal cortical areas and the hippocampus—structures previously implicated in ketamine’s antidepressant effects^29^. Furthermore, our studies provide direct evidence of a ketamine-induced 3 Hz oscillation in posteromedial cortex that has been proposed as a mechanism for its dissociative effects^30^. By analyzing changes in neural oscillations after the addition of propofol in 7 out of 10 subjects, we were also able to identify putative NMDA-mediated brain dynamics that could be antagonized by propofol’s GABAergic activity, as well as possible HCN1-mediated effects where both drugs showed an additive effect. Overall, our results suggest that ketamine engages different neural circuits in distinct frequency-dependent patterns of activity to produce its antidepressant and dissociative sensory effects. These insights may help guide the development of brain dynamic biomarkers and novel therapeutics for depression.

**Fig. 5:**
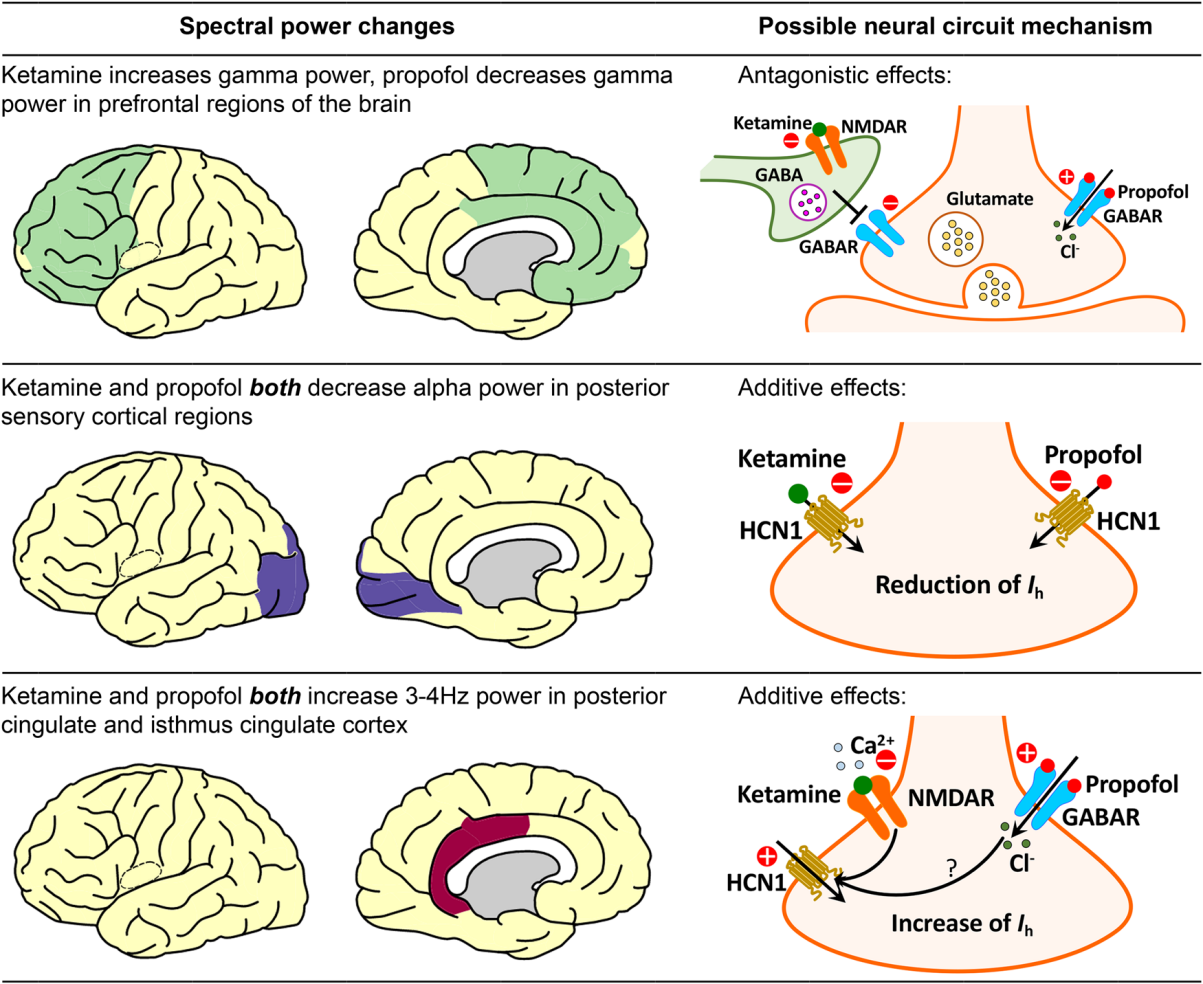
Possible neural circuit mechanisms for subanesthetic dose of ketamine and propofol-induced spectral power changes studied here (Please refer to Zanos et al. , 2018 for a more comprehensive view of the possible mechanisms of ketamine’s action as an antidepressant^51^. This figure was produced using the Simple-brain-plot MATLAB function^66^ and stock drawings of neurons and receptors from Motifolio^67^). NMDAR: N-methyl-D-aspartate receptor; GABA: gamma-aminobutyric acid; GABAR: gamma-aminobutyric acid receptor; HCN1: hyperpolarization-activated cyclic nucleotide-gated potassium channel 1; *Ih*: hyperpolarization-activated cationic current.

For gamma frequencies (25–55 Hz), we observed a remarkable increase in power in frontal and limbic structures that are consistent with previous reports employing non-invasive EEG in humans under both subanesthetic and anesthetic doses of ketamine^7,12–14,38^. We found that the gamma band activity was reversed after the subsequent addition of propofol in prefrontal cortical structures. We propose that the ketamine-induced gamma power increase and its subsequent reversal by propofol could be explained by an antagonist mechanism (Fig. 5, top **panel**). Ketamine preferentially blocks the NMDA receptors on GABAergic inhibitory interneurons, resulting in disinhibition of the downstream excitatory pyramidal neurons, which mediates the increased gamma-band activity^19–21^. When propofol, a GABA agonist, is administered alongside ketamine, it antagonizes the gamma power increase by restoring some of the inhibitory activity in the prefrontal cortex. The increase in gamma spectral power anteriorly following subanesthetic ketamine infusion may reflect a shift of brain activity from a globally balanced state to a disorganized and autonomous state^39^. The changes in gamma band activity in sensory cortices may contribute to the discoordination of higher-order functional networks and perceptual distortions produced by subanesthetic doses of ketamine^32,40,41^.

In contrast, for alpha frequencies (8–15 Hz), we detected a large reduction in iEEG power after ketamine infusion for all brain regions studied, with the largest reductions occurring in posterior sensory cortices. When propofol was subsequently administered, the reduction in alpha power was reversed in most brain regions, suggesting a similar NMDA-dependent mechanism as described above for gamma activity. However, in posterior sensory structures (lingual, pericalcarine and occipital cortices), the addition of propofol further attenuated alpha power. We attribute this additive behavior to ketamine and propofol’s shared inhibition of HCN1 channels (Fig. 5**, middle panel**). HCN1 channels have been identified as an important molecular target for ketamine’s action^24^. Knockout of HCN1 channels abolishes the ketamine-induced loss-of-right reflex, a behavioral correlate of unconsciousness in rodents^24^. Propofol also inhibits HCN1 channels and the HCN1 knock-out mice are known to be less sensitive to unconsciousness due to propofol^24^. Modeling studies suggest that reductions in hyperpolarization-activated cationic current (*Ih*) mediated by HCN1 can abolish occipital alpha rhythms by silencing thalamocortical cells^42^. The reduction of alpha power in occipital regions is also observed during anesthetic doses of ketamine^12,13^, propofol-induced unconsciousness^43^, as well as sleep^44,45^, suggesting the loss of occipital alpha rhythms may be a hallmark for disrupted sensory processing in different states of altered arousal^46^.

We found that subanesthetic doses of ketamine induced a 3 Hz oscillation in PMC in humans, consistent with previous studies in mice after administration of ketamine and in an epileptic patient during a pre-seizure aura as well as in response to electrical stimulation of epileptic foci^30^. Vesuna, et al., 2020, showed that there are NMDA receptors and HCN1 channels in the homologous deep retrosplenial (RSP) cortex in mice, both of which are required for generating the observed 3 Hz rhythmic activity^30^. Knockout of HCN1 channels abolished ketamine-induced rhythms in RSP and the dissociation-related behavior in mice, whereas optogenetic inhibition of long-range inputs to the RSP enhanced ketamine-induced oscillations^30^. Vesuna et al., proposed that ketamine blockade of NMDA receptors could hyperpolarize membrane potentials in PMC, activating intrinsic HCN1 channels and permitting rhythmic dynamics. We propose that the same effect could occur with propofol by way of a GABA-mediated hyperpolarization (Fig. 5**, bottom panel**). Although both ketamine and propofol-induced 3 Hz rhythms in PMC, dissociation was only detected after ketamine. This may be because propofol suppresses arousal and induces unconsciousness, which would supersede any perceived dissociative effects.

Besides its dissociative effects, subanesthetic ketamine has been shown to have a powerful antidepressant effect. The oscillatory circuit dynamics produced by ketamine may be related to this antidepressant effect. Subjects with a history of depression have been observed to have higher amplitude delta and theta oscillations compared to controls during a working memory task^47^. Consistent with this observation, we found that ketamine reduces delta and theta oscillation power. Patients with depression have also been reported to have increased activity in alpha, beta, and theta bands at the occipital and parietal regions of the brain^48^. Accordingly, we identified a global reduction of power at theta, alpha and beta frequencies, with the largest reduction in occipital and parietal regions after ketamine infusion. Gamma oscillations have also been discussed as a potential biomarker for depression. Changes in gamma rhythms can vary according to behavioral states and task conditions, but there are a few studies suggesting that reduced gamma power is associated with depression. One EEG study found that subjects with high depression scores had reduced resting gamma power in the anterior cingulate cortex^49^. Another MEG study showed that depressed subjects with lower baseline gamma and higher ketamine-induced gamma had a better response to ketamine than those with higher baseline gamma^16^. It has also known that the prefrontal cortex and hippocampus are implicated in ketamine’s antidepressant response^29^. The dramatic increase in gamma rhythms we identified in those brain regions with subanesthetic doses of ketamine are consistent with previous studies.

In this study, although we did not directly measure clinical depression nor antidepressant effects, we inferred that our results could be related to ketamine’s antidepressant effects, based on the neuroanatomy of the brain oscillations we identified and prior literature that showed associations among depression, brain dynamics, and functional neuroanatomy. Future studies investigating brain dynamics after ketamine infusion in depressed patients are needed. In this study, we focused primarily on the role of NMDA receptors, which appear to play a central role in mediating ketamine’s effects on brain dynamics^50^ as well as its antidepressant effects^51^. The role of other receptors such as AMPA^52,53^ that have been suggested to play an important role in ketamine’s antidepressant effects should also be investigated in the future. In follow-up studies it would also be interesting to explore the relationship between EEG oscillatory dynamics and the intensity level of dissociation, which could not be addressed in the current study due to our limited sample size and the limited resolution of dissociation assessment. Cross-frequency coupling analysis could be an additional topic of interest for characterizing the interactions between oscillations at different frequency bands. Our results also show how the combination of ketamine and propofol could contribute to unconsciousness through a shared mechanism, providing an explanation for why propofol and ketamine appear to work synergistically to maintain unconsciousness when administered during general anesthesia^54^. Overall, we find that ketamine has distinct dynamic effects on neural systems known to mediate cognition, depression, and sensory processing by way of multiple dissociable neuropharmacological mechanisms. The neural circuit mechanisms underlying ketamine-induced oscillatory dynamics, and their potential links to antidepressive and dissociative effects as proposed in this study, may have important implications for the development of novel therapies with fewer side effects and greater safety.

## Methods

### Subject recruitment

Patients with medication-refractory epilepsy implanted with intracranial depth electrodes to locate their seizure onset zone were recruited from Massachusetts General Hospital and Brigham and Women’s Hospital. Electrode placement was determined by the clinical team independent of this study. Ten patients (five male and five female) aged 22 to 59 years old were recruited. Subjects’ demographic and electrode information are summarized in Table 1. This study was approved by the Institutional Review Board (IRB) covering the two hospitals (Mass General Brigham Human Research Committee). Informed consent was obtained from all subjects prior to the study.

### Experimental procedure

All experiments were conducted during stereotactic neurosurgery for removal of the intracranial depth electrodes in the operating room at the Massachusetts General Hospital or the Brigham and Women’s Hospital. Participants were implanted with multi-lead depth electrodes (a.k.a. stereotactic EEG, sEEG) to confirm the hypothesized seizure focus, and located epileptogenic tissue in relation to essential cortex, thus directing surgical treatment. Depth electrodes (Ad-tech Medical, Racine WI, USA, or PMT, Chanhassen, MN, USA) with diameters of 0.8–1.0 mm and consisting of 8–16 platinum/iridium-contacts 1–2.4 mm long were stereotactically placed in locations deemed necessary for seizure localization by a multidisciplinary clinical team. The first period was a baseline recording of 5 min (Fig. 1). The second period consisted of 14 min with continuous infusion of subanesthetic level of ketamine (total dose of 0.5 mg/kg over 14 min, Supplementary Fig. 7 shows pharmacokinetic effects of different ketamine delivery schemes). At the end of ketamine infusion, a clinical research staff member administered the abbreviated CADSS questionnaire (Supplementary Fig. 2) to the patients^35–37^. Because of limited time in the operating room, patients only answered yes or no to the questions. Immediately after the questionnaire, propofol bolus was given to the patients to induce general anesthesia. During the whole process, subjects were instructed to close their eyes to avoid eye-blink artifacts in the signal. Supplementary Fig. 8 shows oxygen saturation (SpO_2_), mean arterial pressure (MAP), pulse, and end-tidal CO_2_ for the study period. iEEG signals were recorded using a Blackrock Cerebus system (Blackrock Microsystems) sampled at 2,000 Hz. Before each study, structural MRI scans were acquired for each subject (Siemens Trio 3 Tesla, T1-weighted magnetization-prepared rapid gradient echo, 1.3-mm slice thickness, 1.3 × 1 mm in-plane resolution, TR/TE = 2530/3.3 ms, 7° flip angle).

### iEEG preprocessing, power spectral analysis and statistical analysis

Data analysis was performed using custom analysis code in MATLAB (R2021a). Raw iEEG data were notch filtered at 60 Hz and its harmonics, downsampled to 500 Hz, and detrended across the entire recording. The signals were then visually inspected, and channels with noise or artifacts were removed. Data were re-referenced with a bipolar montage. A total of 824 bipolar channels were generated for 10 subjects received ketamine, and 606 bipolar channels were generated for 7 subjects received propofol (Supplementary Fig. 1 and Supplementary Video). Spectral analysis was performed using the multitaper method, with window lengths of T = 2 sec with 0.5 sec overlap, time-bandwidth product TW = 3, number of tapers *K* = 5, and spectral resolution of 3 Hz^55,56^. The mean power spectral density for baseline, ketamine and propofol conditions were calculated by taking the average across each period. The power spectral density was converted to decibels (dB) to facilitate easier comparisons. The differences of power after ketamine infusion relative to baseline, and propofol relative to ketamine periods were calculated by subtracting the mean power spectral density in dB between each of the two conditions at different frequencies (slow: 0.1–1 Hz, delta: 1–4 Hz, theta: 4–8 Hz, alpha: 8–15 Hz, beta: 15–25 Hz, gamma: 25–55 Hz, low gamma: 25–40 Hz, upper gamma: 40–55 Hz). Our primary objective was to describe changes in iEEG power by reporting effect sizes and confidence intervals for changes in iEEG power in the indicated brain regions of interest (ROIs) after drug administration. We did not report p-values and thus did not correct for multiple comparisons. The bootstrap method was used to generate the 95% confidence interval around the mean differences in power for each structural label at each frequency using data from all subjects who had electrodes located within each structural label. The upper and lower bars represent the bootstrapped 95% confidence interval bounds.

### Structural parcellation of the brain

The electrode positions in each subject’s brain were obtained by aligning the preoperative T1-weighted MRI with a postoperative CT/MRI using the Freesurfer (7.2) image analysis tool^57,58^. To identify the structural label and functional network for each of the electrodes, an electrode labeling algorithm (ELA) was employed^59^. This algorithm estimated the probability of overlap of an expanding area around each electrode with brain structural labels that had been identified in the Desikan-Killiany-Tourville (DKT) 40 atlas using purely anatomical approaches^60–65^. Then the ELA used gradient descent to find the closest voxel in the template’s brain that gives similar regions and probabilities to transform the patients’ electrode coordinates to the template brain^60–64^. Based on DKT 40 atlas, we assigned the 824 electrodes from 10 subjects received ketamine to 49 structural labels, which were then further classified into 15 labels according to the anatomical locations and the mean differences of power after ketamine relative to the baseline condition. Likewise, we assigned the 606 electrodes collected from 7 subjects received propofol to 14 structural labels. We plotted all electrodes on Colin 27 template brain with colors per parcellated brain region indicating the differences in power for the ketamine infusion period relative to baseline, as well as for propofol bolus relative to the ketamine infusion period for each of the frequencies.

### Reporting summary

Further information on research design is available in the Nature Portfolio Reporting Summary linked to this article.

## Supplementary information

Supplementary Information

Peer Review File

Description of Additional Supplementary Files

Supplementary Movie 1

Reporting Summary

## Data Availability

The data that support the findings of this study are available on request from the corresponding author. The raw data are not publicly available due to restrictions relating to the per-participant imaging data currently containing information that could compromise the privacy of research participants. The DKT 40 classifier atlas are available at https://mindboggle.info/data. Source data are provided with this paper.

## Source data

Source Data

## Code availability

The codes that support the findings of this study are available from the corresponding author upon request.
