# Supplementary material for: Characterizing brain dynamics during ketamine-induced dissociation and subsequent interactions with propofol using human intracranial neurophysiology
Source: Nat Commun. 2023 Mar 29;14:1748. doi: 10.1038/s41467-023-37463-3 (PMC10060225; doi:10.1038/s41467-023-37463-3)
Supplement: Supplementary file 1 — Supplementary Information [file 41467_2023_37463_MOESM1_ESM.pdf]

Supplementary Table 1. Response to questionnaire

Subject ID	1	2	3	4	5	6	7	8	9	10
Question 1	Yes	No	Yes	Yes	NR	Yes	Yes	Yes	Yes	Yes
Question 2	Yes	Yes	Yes	Yes	Yes	No	Yes	NR	Yes	Yes
Question 3	Yes	Yes	Yes	NR	NR	No	Yes	NR	Yes	AR
Question 4	Yes	Yes	Yes	Yes	No	No	Yes	Yes	Yes	No
Question 5	Yes	Yes	Yes	NR	NR	No	Yes	Yes	Yes	No
Question 6	No	Yes	Yes	Yes	Yes	No	Yes	Yes	Yes	No
Question 7	NR	Yes	Yes	NR	Yes	Yes	No	Yes	No	No
Question 8	Yes	Yes	Yes	NR	Yes	No	Yes	Yes	Yes	No
Question 9	Yes	Yes	Yes		AR	No	Yes	Yes	Yes	No
Question 10	Yes	No	Yes	NR		No	No	Yes	No	No
Question 11	NR	No	No	NR		No	No	Yes	No	No
Question 12	No	No	No	NR		Yes	No	Yes	No	No
Question 13	No	No	Yes			Yes	No	Yes	Yes	No
Question 14	No	Yes	No			No	Yes	Yes	No	No
Question 15		Yes	No	Yes	Yes	No	Yes	Yes	Yes	No
Question 16		Yes	No	NR	NR	No	No	Yes	Yes	No
Question 17		Yes	Yes	NR	AR	No	No	Yes	Yes	No
Question 18		Yes	Yes	NR	Yes	No	Yes	Yes	Yes	No
Question 19		Yes	Yes	NR	NR	No	Yes	Yes	No	No
Question 20		Yes	Yes		NR	Yes	Yes	Yes	Yes	No
Question 21		No	No		NR	No	Yes	Yes	Yes	No
Question 22		Yes	Yes		Yes	No	Yes	Yes	Yes	No
Question 23		No	No		No	No	Yes	Yes	No	No
Number of Yes (%)	8 (57.1)	16 (69.6)	16 (69.6)	5 (31.2)	7 (38.9)	5 (21.7)	16 (69.6)	21 (91.3)	16 (69.6)	2 (8.7)
Number of No (%)	4 (28.6)	7 (30.4)	7 (30.4)	0 (0)	2 (11.1)	18 (78.3)	7 (30.4)	0 (0)	7 (30.4)	20 (87.0)
Number of NR/AR (%)	2 (14.3)	0 (0)	0 (0)	11 (68.8)	9 (50.0)	0 (0)	0 (0)	2 (8.7)	0 (0)	1 (4.3)
Total	14*	23	23	16*	18*	23	23	23	23	23

NR: no response; AR: ambiguous response. *We did not finish all the questions for this patient because of limited time in the operating room

Supplementary Table 2. Mean and bootstrap 95% confidence interval for intracranial EEG power changes after ketamine infusion relative to baseline at 6 frequencies for 15 structural labels

Structural Labels (N=10, n=824)	Slow (0.1-1Hz)	Delta (1-4Hz)	Theta (4-8Hz)	Alpha (8-15Hz)	Beta (15-25Hz)	Gamma (25-55Hz)
Anterior and Posterior Cingulate (N=7, n=55)	-2.06 [-2.96, -1.26]	-3.88 [-6.19, -1.71]	2.41 [-0.05, 4.88]	-14.33 [-19.52, -9.11]	4.49 [-2.58, 12.15]	159.04 [134.42, 189.31]
Superior Frontal (N=7, n=23)	-2.27 [-3.87, -0.82]	-6.67 [-10.74, -2.83]	-5.65 [-11.94, -1.15]	-24.11 [-37.22, -11.36]	0.54 [-13.91, 16.47]	153.03 [115.95, 204.18]
Middle Frontal (N=7, n=79)	-2.80 [-3.97, -1.86]	-4.93 [-7.97, -2.30]	0.02 [-4.18, 3.88]	-26.48 [-33.50, -20.14]	-14.26 [-21.34, -6.56]	153.59 [130.80, 181.64]
Orbitofrontal (N=7, n=49)	0.25 [-0.87, 1.65]	-2.83 [-5.27, 0.23]	0.63 [-1.34, 2.58]	-7.14 [-11.21, -3.14]	2.17 [-2.56, 7.72]	133.68 [113.17, 162.77]
Inferior Frontal (N=9, n=56)	-2.54 [-4.36, -1.25]	-7.92 [-11.72, -5.04]	-0.44 [-3.39, 2.47]	-11.83 [-16.42, -8.10]	4.63 [-0.01, 10.28]	149.20 [126.44, 175.51]
Precentral (N=5, n=18)	-2.44 [-5.67, -0.87]	-8.09 [-15.67, -3.74]	-9.96 [-23.05, -2.58]	-26.50 [-53.55, -12.97]	-18.21 [-24.41, -12.16]	91.70 [64.17, 138.70]
Postcentral (N=3, n=23)	-1.74 [-3.23, -0.50]	-4.68 [-7.45, -2.18]	-4.50 [-7.76, -1.03]	-33.55 [-40.24, -25.96]	-36.00 [-44.52, -26.68]	72.47 [48.84, 96.83]
Isthmus Cingulate (N=4, n=25)	-0.61 [-1.50, 0.22]	-0.08 [-1.77, 1.36]	1.44 [-2.08, 5.94]	-13.90 [-17.34, -9.92]	-10.53 [-14.76, -6.99]	43.09 [34.43, 53.46]
Parietal, Precuneus and Supramarginal (N=4, n=45)	-2.40 [-2.99, -1.93]	-4.84 [-6.61, -3.55]	-7.75 [-10.71, -5.66]	-14.94 [-18.37, -12.23]	-15.90 [-20.59, -11.49]	5.15 [-9.49, 15.96]
Temporal and Fusiform (N=10, n=260)	-2.34 [-2.71, -2.02]	-5.37 [-6.23, -4.63]	-5.83 [-7.39, -4.41]	-12.02 [-13.36, -10.76]	-8.40 [-10.37, -6.17]	28.87 [22.35, 36.81]
Lingual and Pericalcarine (N=4, n=39)	-1.64 [-2.49, -0.77]	-6.84 [-8.63, -5.18]	-11.68 [-15.10, -7.64]	-27.02 [-31.36, -23.23]	-19.58 [-24.61, -13.98]	19.07 [9.21, 30.70]
Occipital (N=2, n=18)	-3.51 [-4.29, -2.30]	-12.91 [-15.15, -10.13]	-20.52 [-23.12, -17.76]	-32.07 [-38.12, -24.93]	-43.81 [-48.21, -38.54]	-42.96 [-68.30, -18.75]
Hippocampus and Amygdala (N=9, n=113)	-1.51 [-1.96, -0.95]	-3.91 [-5.02, -2.36]	-0.25 [-2.09, 1.54]	-1.54 [-3.81, 0.83]	4.43 [1.69, 7.64]	68.64 [56.44, 82.26]
Striatum (N=3, n=4)	0.62 [-2.51, 3.01]	-1.40 [-2.63, -0.16]	-1.85 [-2.94, -0.12]	-26.60 [-32.82, -17.48]	-2.47 [-10.32, 10.23]	96.37 [53.39, 119.42]
Insula (N=4, n=17)	-0.23 [-1.17, 0.49]	0.13 [-2.49, 2.02]	3.88 [1.42, 6.56]	-5.44 [-8.42, -2.47]	1.46 [-2.57, 7.51]	56.30 [41.29, 75.39]

N: number of subjects for each structural label; n: number of electrodes for each structural label

Supplementary Table 3. Mean and bootstrap 95% confidence interval for intracranial EEG power changes after propofol bolus relative to ketamine period at 7 frequencies for 14 structural labels

Structural Labels (N=7, n=606)	Slow (0.1-1Hz)	Delta (1-4Hz)	Theta (4-8Hz)	Alpha (8-15Hz)	Beta (15-25Hz)	Low Gamma (25-40Hz)	Upper Gamma (40-55Hz)
Anterior and Posterior Cingulate (N=4, n=30)	38.69 [33.40, 41.91]	87.25 [74.65, 95.27]	60.54 [50.71, 68.26]	144.08 [128.50, 158.41]	145.45 [116.86, 167.21]	-16.45 [-71.27, 29.72]	-61.26 [-99.98, -22.67]
Superior Frontal (N=4, n=14)	33.66 [29.40, 37.45]	77.24 [67.49, 86.04]	52.74 [46.16, 59.77]	124.91 [99.21, 152.48]	108.56 [68.59, 162.20]	-32.50 [-119.83, 44.73]	-68.32 [-146.89, -17.11]
Middle Frontal (N=4, n=43)	38.66 [35.20, 41.59]	92.64 [86.29, 97.80]	57.42 [51.52, 62.36]	113.78 [99.02, 128.28]	114.66 [91.13, 136.67]	-68.95 [-118.38, -21.95]	-134.51 [-177.93, -96.62]
Orbitofrontal (N=6, n=43)	30.58 [26.17, 34.75]	69.38 [59.03, 78.04]	51.24 [44.36, 57.60]	137.43 [121.60, 150.88]	166.62 [139.86, 190.57]	44.88 [-0.68, 82.50]	-52.27 [-93.71, -20.62]
Inferior Frontal (N=6, n=39)	25.16 [20.11, 29.67]	58.60 [46.56, 68.51]	39.23 [31.56, 46.41]	110.97 [91.94, 127.35]	155.03 [124.18, 181.32]	27.40 [-16.10, 63.85]	-61.86 [-107.45, -24.86]
Precentral (N=4, n=17)	25.03 [20.92, 30.05]	65.87 [58.90, 74.49]	63.91 [59.31, 70.28]	158.32 [141.96, 179.07]	175.00 [136.46, 212.71]	136.59 [80.92, 197.51]	49.39 [12.05, 91.84]
Postcentral (N=2, n=5)	18.30 [14.43, 23.12]	57.76 [49.72, 66.49]	68.88 [64.37, 78.00]	154.79 [127.73, 179.99]	214.90 [186.07, 241.30]	170.25 [135.60, 223.74]	67.34 [32.12, 97.19]
Isthmus Cingulate (N=3, n=20)	20.69 [17.61, 24.98]	33.62 [23.21, 45.70]	8.12 [-6.46, 21.40]	33.33 [14.49, 54.32]	98.33 [79.28, 117.16]	86.48 [73.32, 99.47]	9.78 [0.02, 17.19]
Parietal, Precuneus and Supramarginal (N=3, n=35)	26.19 [23.53, 28.48]	55.35 [46.56, 62.83]	22.58 [14.76, 30.52]	34.81 [23.51, 49.37]	82.40 [72.07, 95.51]	56.20 [47.10, 64.57]	4.06 [-2.04, 10.02]
Temporal and Fusiform (N=7, n=206)	24.00 [22.42, 25.64]	42.53 [38.55, 46.45]	23.13 [19.15, 26.86]	45.74 [39.17, 52.19]	97.43 [88.09, 106.81]	64.81 [53.53, 75.52]	1.33 [-9.12, 10.38]
Lingual and Pericalcarine (N=3, n=33)	19.33 [16.72, 22.27]	33.42 [25.40, 41.48]	1.87 [-8.90, 12.02]	-15.66 [-38.74, 4.61]	12.61 [-5.12, 29.70]	44.45 [33.00, 55.02]	-4.32 [-15.44, 6.87]
Occipital (N=2, n=18)	24.75 [21.21, 29.22]	43.60 [33.34, 55.91]	-3.32 [-13.56, 8.03]	-35.20 [-58.21, -14.02]	22.00 [1.95, 42.76]	60.47 [50.09, 71.30]	18.85 [11.40, 26.00]
Hippocampus and Amygdala (N=7, n=90)	20.87 [18.74, 23.05]	43.87 [39.02, 48.67]	43.99 [38.04, 49.77]	111.87 [100.95, 122.29]	151.28 [137.11, 164.07]	108.87 [91.29, 124.79]	20.56 [4.73, 34.94]
Insula (N=3, n=13)	7.31 [4.67, 11.50]	10.29 [0.37, 21.78]	17.35 [4.97, 30.18]	116.90 [99.23, 142.82]	124.26 [105.76, 149.45]	73.51 [52.86, 98.92]	8.17 [-0.07, 20.66]

N: number of subjects for each structural label; n: number of electrodes for each structural label

Supplementary Table 4. Mean and bootstrap 95% confidence interval for 3-4 Hz intracranial EEG power changes after administration of ketamine and propofol

Structural Labels	Ketamine vs. Baseline (N=10, n=824)	Propofol vs. Ketamine (N=7, n=606)
Rostral Anterior Cingulate	-0.33 [-1.52, 0.88] (N=7, n=26)	18.48 [11.64, 23.45] (N=4, n=13)
Caudal Anterior Cingulate	0.35 [-0.81, 1.57] (N=6, n=25)	23.62 [20.37, 25.57] (N=3, n=13)
Superior Frontal	-1.51 [-2.87, -0.25] (N=7, n=23)	19.56 [16.92, 22.11] (N=4, n=14)
Rostral Middle Frontal	0.86 [-0.70, 2.62] (N=7, n=57)	24.01 [22.29, 25.43] (N=4, n=32)
Caudal Middle Frontal	-0.53 [-2.88, 1.08] (N=6, n=22)	24.57 [22.26, 26.37] (N=3, n=11)
Lateral Orbitofrontal	-0.98 [-1.61, -0.41] (N=7, n=39)	17.18 [14.11, 19.81] (N=6, n=33)
Medial Orbitofrontal	0.34 [-1.89, 2.99] (N=6, n=10)	18.27 [11.78, 23.27] (N=6, n=10)
Pars Opercularis	2.90 [0.60, 5.82] (N=4, n=10)	4.58 [-1.02, 7.92] (N=2, n=3)
Pars Orbitalis	-2.86 [-5.30, -1.68] (N=5, n=7)	19.07 [16.75, 20.93] (N=4, n=6)
Pars Triangularis	-2.25 [-3.54, -1.33] (N=7, n=39)	14.86 [11.19, 17.85] (N=5, n=30)
Precentral	-1.84 [-4.61, -0.21] (N=5, n=18)	17.70 [16.38, 19.46] (N=4, n=17)
Postcentral	-0.22 [-0.81, 0.32] (N=3, n=23)	18.37 [16.05, 20.68] (N=2, n=5)
Posterior Cingulate	2.05 [0.48, 3.61] (N=1, n=4)	24.18 [23.47, 25.36] (N=1, n=4)
Isthmus Cingulate	1.00 [0.24, 1.90] (N=4, n=25)	6.58 [2.75, 10.29] (N=3, n=20)
Inferior Parietal	-0.19 [-1.01, 0.60] (N=3, n=24)	10.22 [5.90, 14.11] (N=2, n=18)
Superior Parietal	-2.45 [-4.41, -1.03] (N=1, n=9)	18.72 [16.92, 20.16] (N=1, n=9)
Precuneus	-1.60 [-2.92, -0.48] (N=1, n=5)	15.30 [14.40, 17.37] (N=1, n=5)
Supramarginal	0.71 [-0.72, 2.10] (N=3, n=7)	8.80 [1.83, 21.03] (N=2, n=3)
Inferior Temporal	-1.68 [-2.14, -1.23] (N=6, n=29)	9.95 [4.82, 13.90] (N=5, n=24)
Superior Temporal	0.16 [-0.32, 0.64] (N=10, n=74)	8.04 [5.86, 10.21] (N=7, n=51)
Middle Temporal	-1.82 [-2.23, -1.45] (N=10, n=141)	9.65 [8.01, 11.26] (N=7, n=115)
Fusiform	-0.28 [-1.34, 0.43] (N=4, n=10)	6.34 [1.64, 10.35] (N=4, n=10)
Transverse Temporal	0.19 [-0.53, 1.49] (N=2, n=6)	2.39 [-1.20, 5.81] (N=2, n=6)
Lingual	-2.31 [-3.29, -1.28] (N=4, n=33)	6.52 [3.35, 9.71] (N=3, n=27)
Pericalcarine	-1.56 [-2.25, -1.10] (N=1, n=6)	6.93 [3.48, 9.69] (N=1, n=6)
Lateral Occipital	-4.49 [-5.21, -3.76] (N=2, n=18)	8.36 [5.10, 11.85] (N=2, n=18)
Hippocampal	-0.70 [-1.13, -0.24] (N=9, n=72)	13.70 [11.53, 15.75] (N=7, n=58)
Amygdala	-0.95 [-1.49, -0.03] (N=8, n=41)	8.08 [6.06, 10.19] (N=6, n=32)
Caudate	-1.73 [-1.81, -1.65] (N=1, n=2)	NA
Putamen	0.04 [-0.46, 0.53] (N=2, n=2)	NA
Insula	0.89 [-0.11, 1.66] (N=4, n=17)	0.95 [-3.11, 4.89] (N=3, n=13)

N: number of subjects for each structural label; n: number of electrodes for each structural label; NA: not applicable

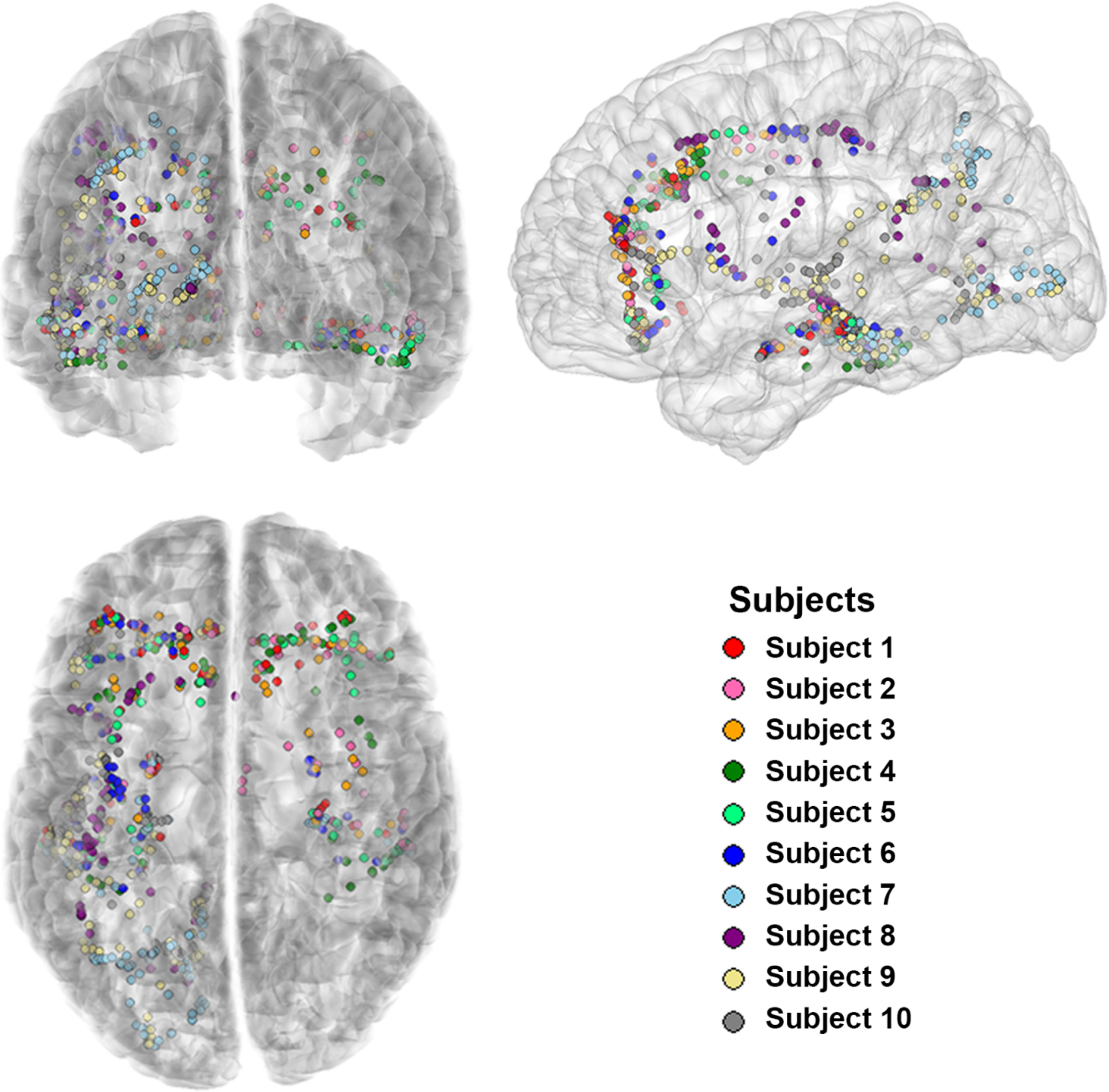

Supplementary Figure 1. Coregistered and reconstructed intracranial electrodes from all subjects on Colin 27 brain template

Abbreviated CADSS

Name___________________ ID______________ Date_______________

Important - general:
(1) Do things seem to be moving in slow motion?
(2) Do things seem to be unreal to you, as if you are in a dream?
(3) Have sounds almost disappeared or become much stronger than you would have expected?
(4) Do you feel as if you are looking at things from outside of your body?
(5) Do you feel as if you are watching the situation as an observer or a spectator?
(6) Do you feel disconnected from your own body?
(7) Does your sense of your own body feel changed: for instance, does your own body feel
unusually large or unusually small?
(8) Do things seem to be happening very quickly, as if there is a lifetime in a moment?
(9) Do you feel like there are different parts of yourself which do not fit together?

Important - visual:
(10) Do objects look different than you would expect?
(11) Do colors seem to be diminished in intensity?
(12) Do you see things as if you were in a tunnel, or looking through a wide angle photographic lens?
(13) Do colors seem much brighter than you would have expected?
(14) Does it seem as if you are looking at the world through a fog, so that people and objects appear far away or unclear?

Less important:
(15) Do you have some experience that separates you from what is happening; for instance, do you feel as if you are in a movie or a play, or as if you are a robot?
(16) Do people seem motionless, dead, or mechanical?
(17) Does this interview [assessment, questionnaire] seem to be taking much longer than you would have expected?
(18) Have there been things which have happened during this interview [assessment] that now you can't account for?
(19) Have you spaced out, or in some other way lost track of what was going on during this experience?
(20) Do things seem very real, as if there is a special sense of clarity?
(21) Do you feel confused about who you really are?
(22) Do you have gaps in your memory?
(23) Do you feel like you have more than one identity?

Supplementary Figure 2. Abbreviated version of the Clinician-Administered Dissociative States Scale (CADSS). A clinical research staff member administered questions to patients. Patients answered yes or no to each question.

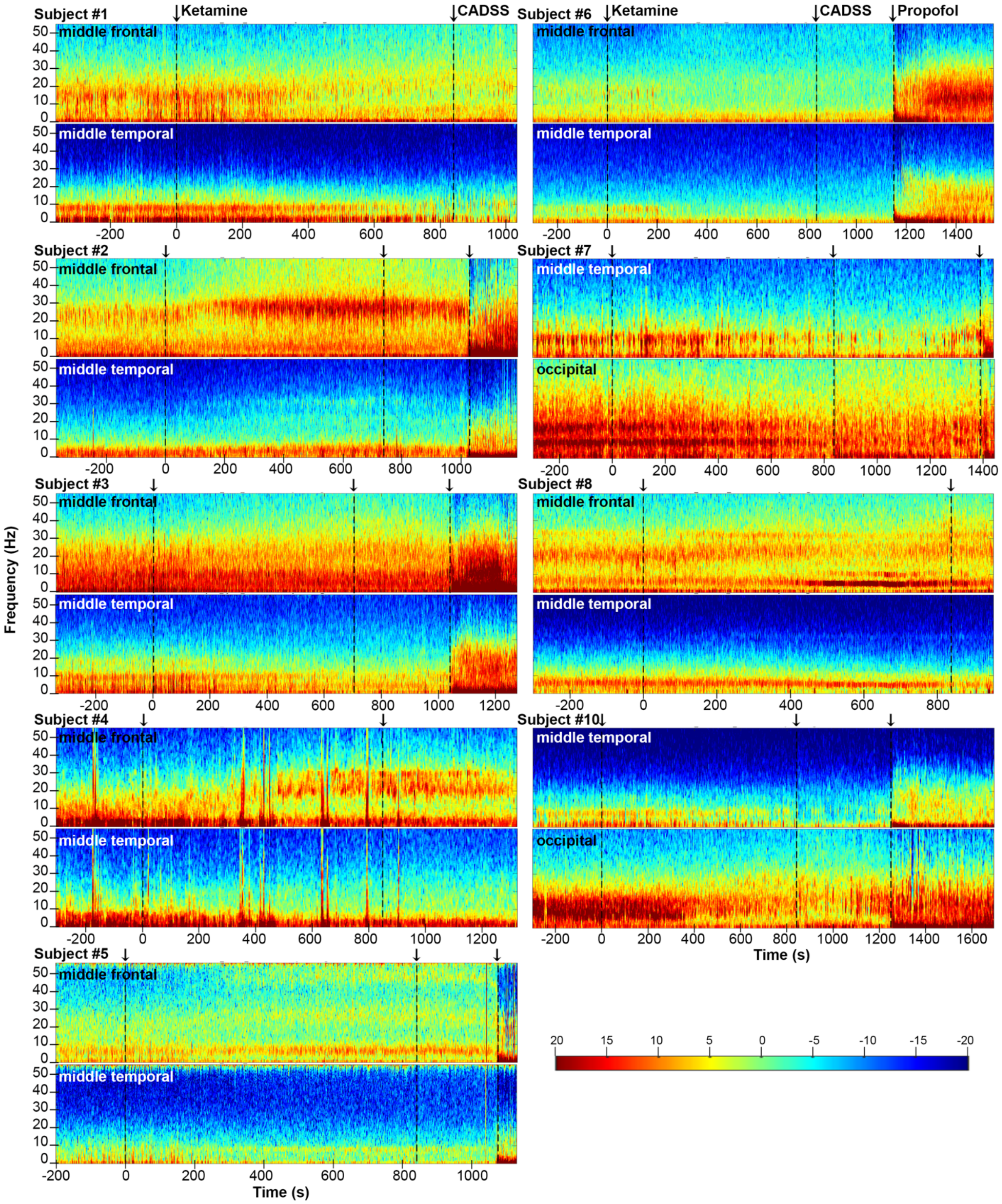

Supplementary Figure 3. Power spectrogram (dB) for frontal, temporal, and occipital channels from individual subject during baseline, ketamine, and propofol conditions. First arrows (0 sec) indicate the start time for ketamine infusion, second arrows indicate the start time for CADSS (Clinician-Administered Dissociative States Scale), and third arrows (if there is) indicate the start time for propofol administration.

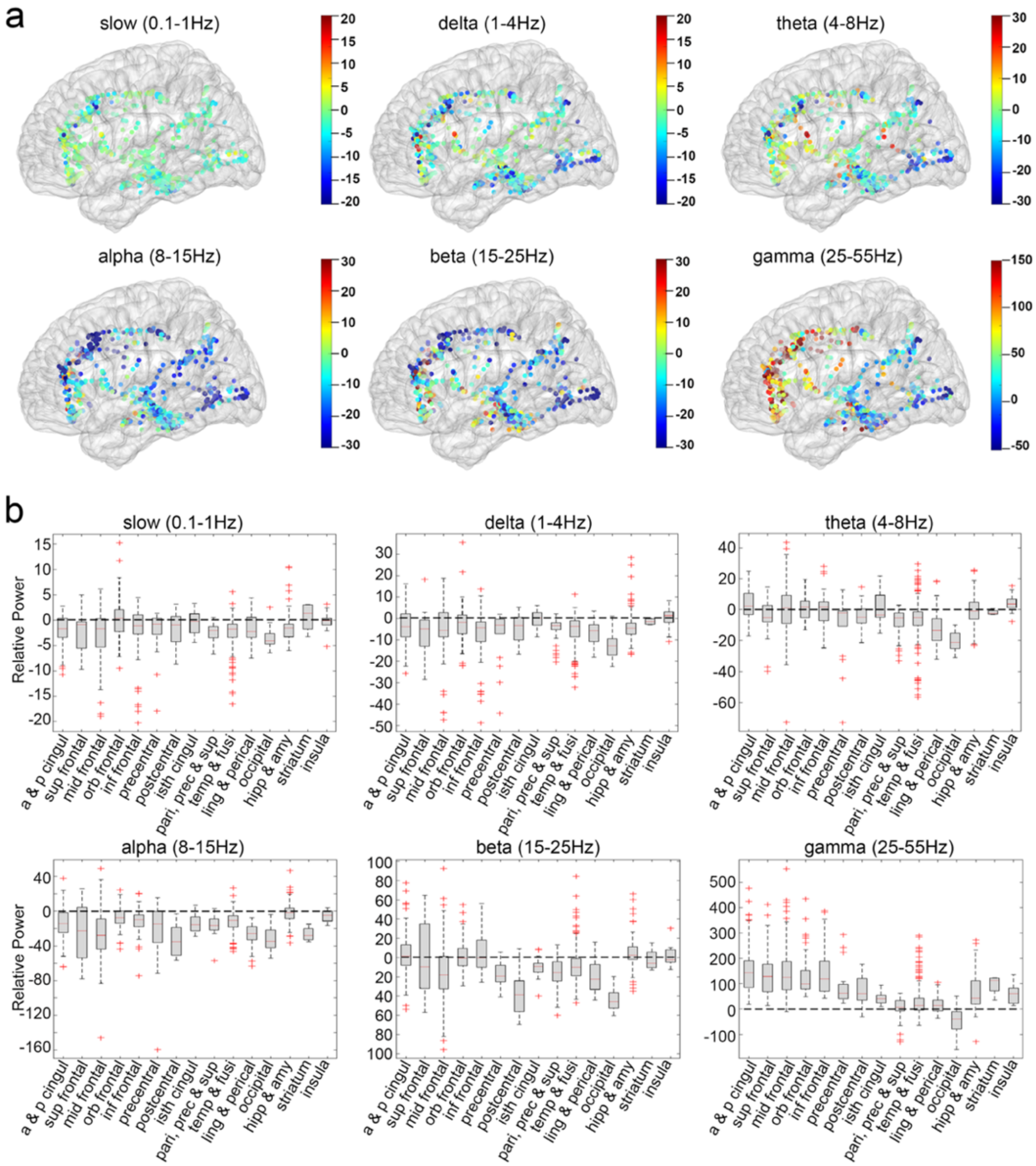

Supplementary Figure 4. Structural mapping for intracranial EEG power changes after ketamine infusion relative to baseline at 6 frequencies, shown in an alternative view depicting intracranial EEG power changes at individual electrode locations. **a,** The mean differences in power (dB) after ketamine infusion relative to baseline were calculated for 824 channels across 10 subjects and plotted on Colin 27 brain template. Warmer colors indicate an increase in power and cooler colors indicate a decrease in power. **b,** Boxplot for intracranial EEG power changes after ketamine infusion relative to baseline at 6 frequencies for 15 structural labels: a & p cingul (anterior and posterior cingulate), sup frontal (superior frontal), mid frontal (middle frontal), orb frontal (orbitofrontal), inf frontal (parsopercularis, parsorbitalis, and parstriangularis), precentral, postcentral, isth cingul (isthmuscingulate), pari, prec & sup (parietal, precuneus, and supramarginal), temp & fusi (temporal and fusiform), ling & perical (lingual and pericalcarine), occipital, hipp & amy (hippocampal and amygdala), striatum (caudate and putamen), and insula. Central mark indicates the median. The bottom and top edges of the box indicate the 25th and 75th percentiles, respectively. The whiskers extend to the most extreme data points not considered outliers. The outliers are plotted individually using the '+' marker symbol.

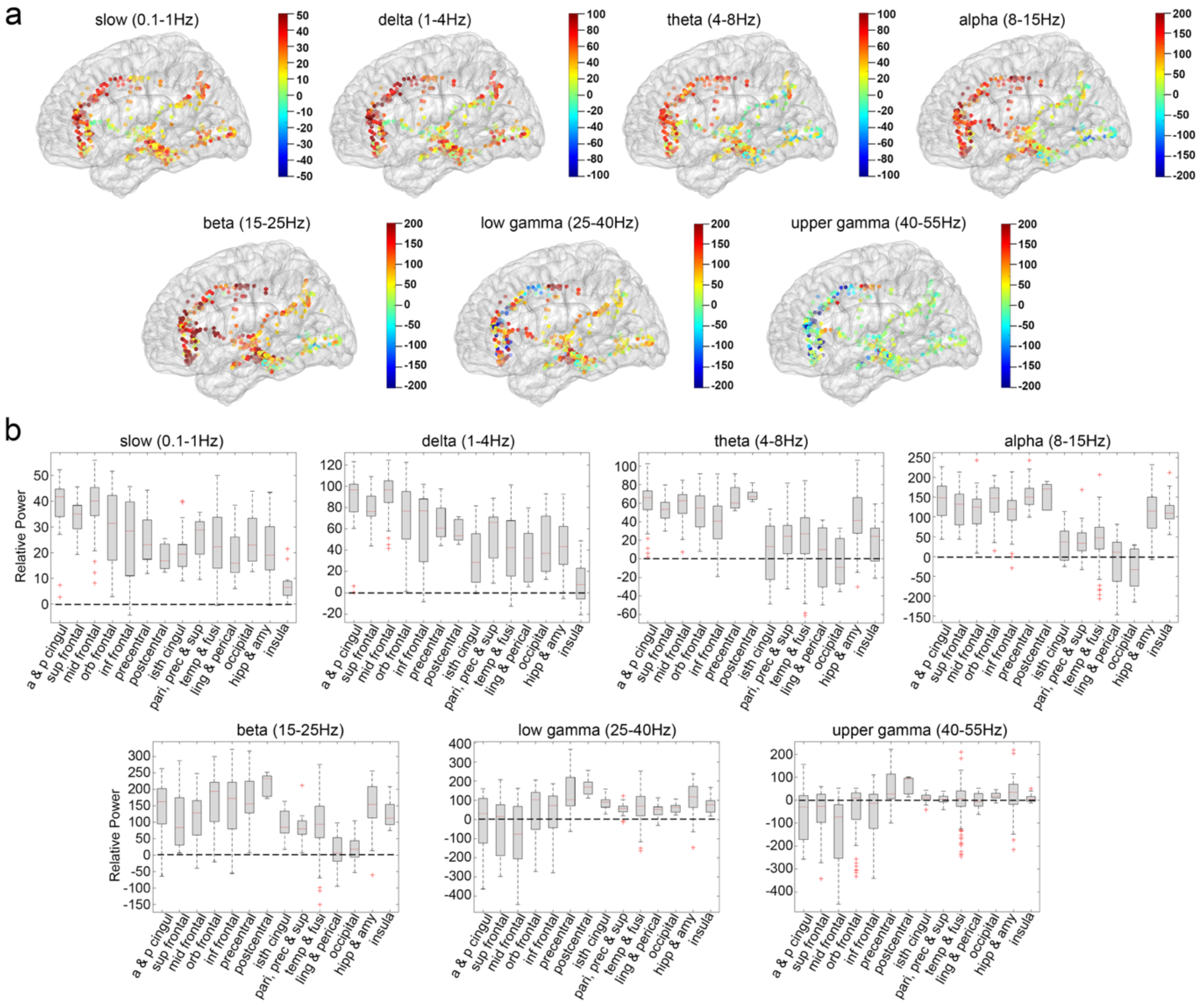

Supplementary Figure 5. Structural mapping for intracranial EEG power changes after propofol bolus relative to ketamine period at 7 frequencies, shown in an alternative view depicting intracranial EEG power changes at individual electrode locations. **a,** The mean differences in power (dB) after propofol bolus relative to ketamine period were calculated for 606 channels across 7 subjects and plotted on Colin27 brain template. Warmer colors indicate an increase in power and cooler colors indicate a decrease in power. **b,** Boxplot for intracranial EEG power changes after propofol bolus relative to ketamine period at 7 frequencies for 14 structural labels: a & p cingul (anterior and posterior cingulate), sup frontal (superior frontal), mid frontal (middle frontal), orb frontal (orbitofrontal), inf frontal (parsopercularis, parsorbitalis, and parstriangularis), precentral, postcentral, isth cingul (isthmuscingulate), pari, prec & sup (parietal, precuneus, and supramarginal), temp & fusi (temporal and fusiform), ling & perical (lingual and pericalcarine), occipital, hipp & amy (hippocampal and amygdala), and insula. Central mark indicates the median. The bottom and top edges of the box indicate the 25th and 75th percentiles, respectively. The whiskers extend to the most extreme data points not considered outliers. The outliers are plotted individually using the '+' marker symbol.

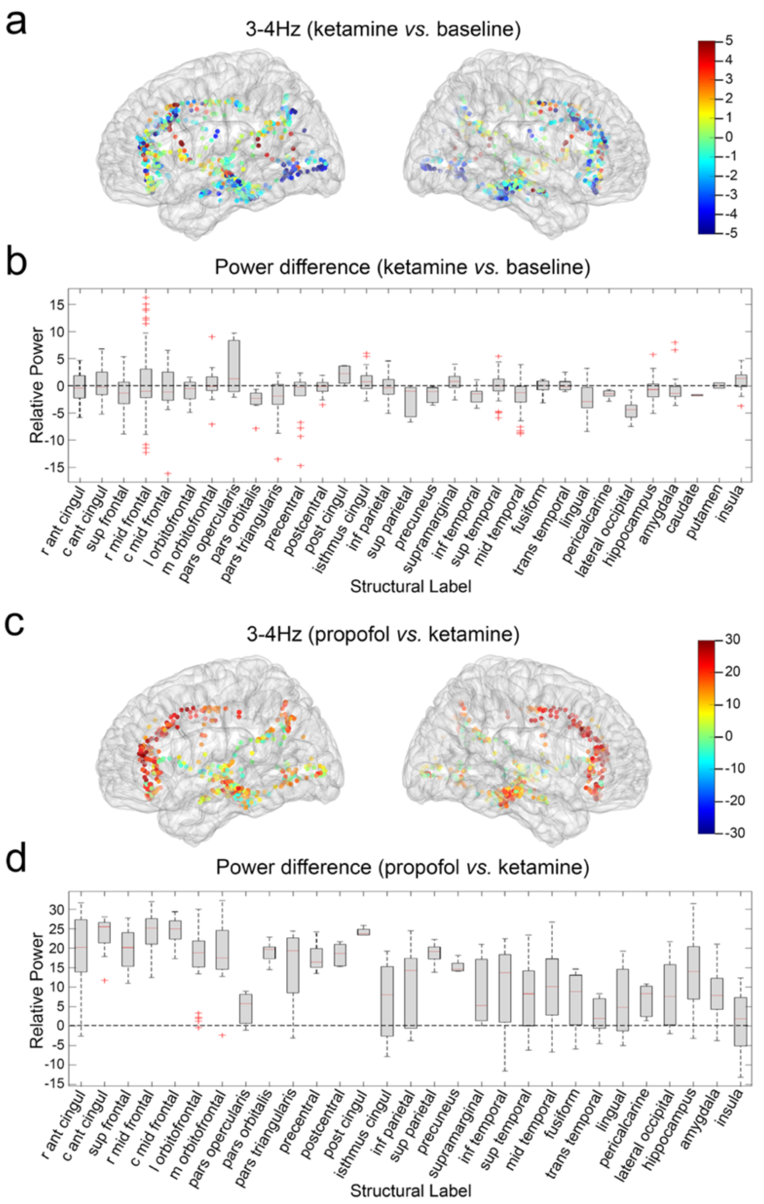

Supplementary Figure 6. Analysis of 3-4 Hz intracranial EEG power changes after administration of ketamine and propofol, shown in an alternative view depicting intracranial EEG power changes at individual electrode locations. **a,** Structural mapping for 3-4 Hz intracranial EEG power (dB) changes after ketamine infusion. **b,** Boxplot for 3-4 Hz intracranial EEG power (dB) changes after ketamine infusion. **c,** Structural mapping for 3-4 Hz intracranial EEG power (dB) changes after propofol bolus. **d,** Boxplot for 3-4 Hz intracranial EEG power (dB) changes after propofol bolus. Central mark indicates the median. The bottom and top edges of the box indicate the 25th and 75th percentiles, respectively. The whiskers extend to the most extreme data points not considered outliers. The outliers are plotted individually using the '+' marker symbol. r ant cingul: rostral anterior cingulate; c ant cingul: caudal anterior cingulate; sup frontal: superior frontal; r mid frontal: rostral middle frontal; c mid frontal: caudal middle frontal; l orbitofrontal: lateral orbitofrontal; m orbitofrontal: medial orbitofrontal; post cingul: posterior cingulate; isthmus cingul: isthmus cingulate; inf parietal: inferior parietal; inf temporal: inferior temporal; sup temporal: superior temporal; mid temporal: middle temporal; trans temporal: transverse temporal.

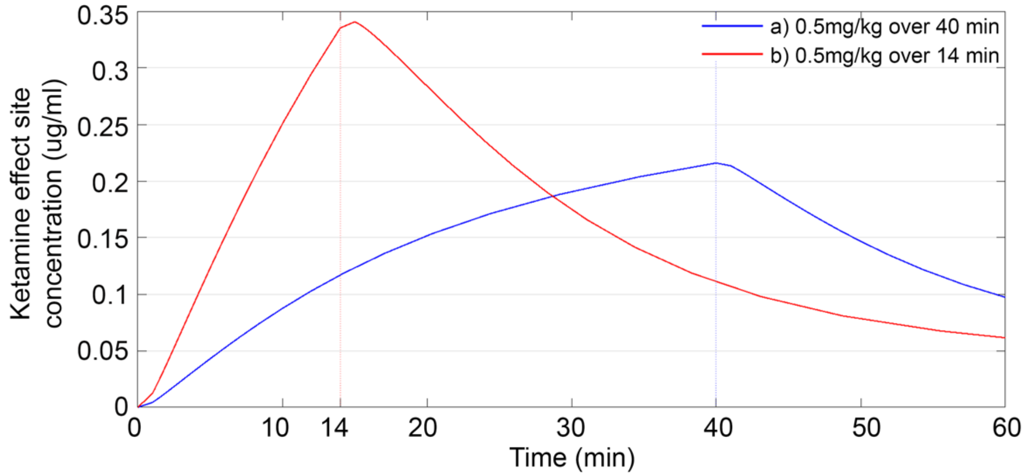

Supplementary Figure 7. Pharmacokinetic effects of different ketamine delivery schemes. a) Infusion of 0.5 mg/kg ketamine over 40 min. b) Infusion of 0.5 mg/kg ketamine over 14 min. Simulations were conducted for a hypothetical 60 kg female participant using pharmacokinetic/ pharmacodynamic (PK/PD) modeling implemented in StanpumpR (https://github.com/StevenLShafer/stanpumpR). Ketamine delivery started at 0 min. Source data are provided as a Source Data file Supplementary Figure 7.

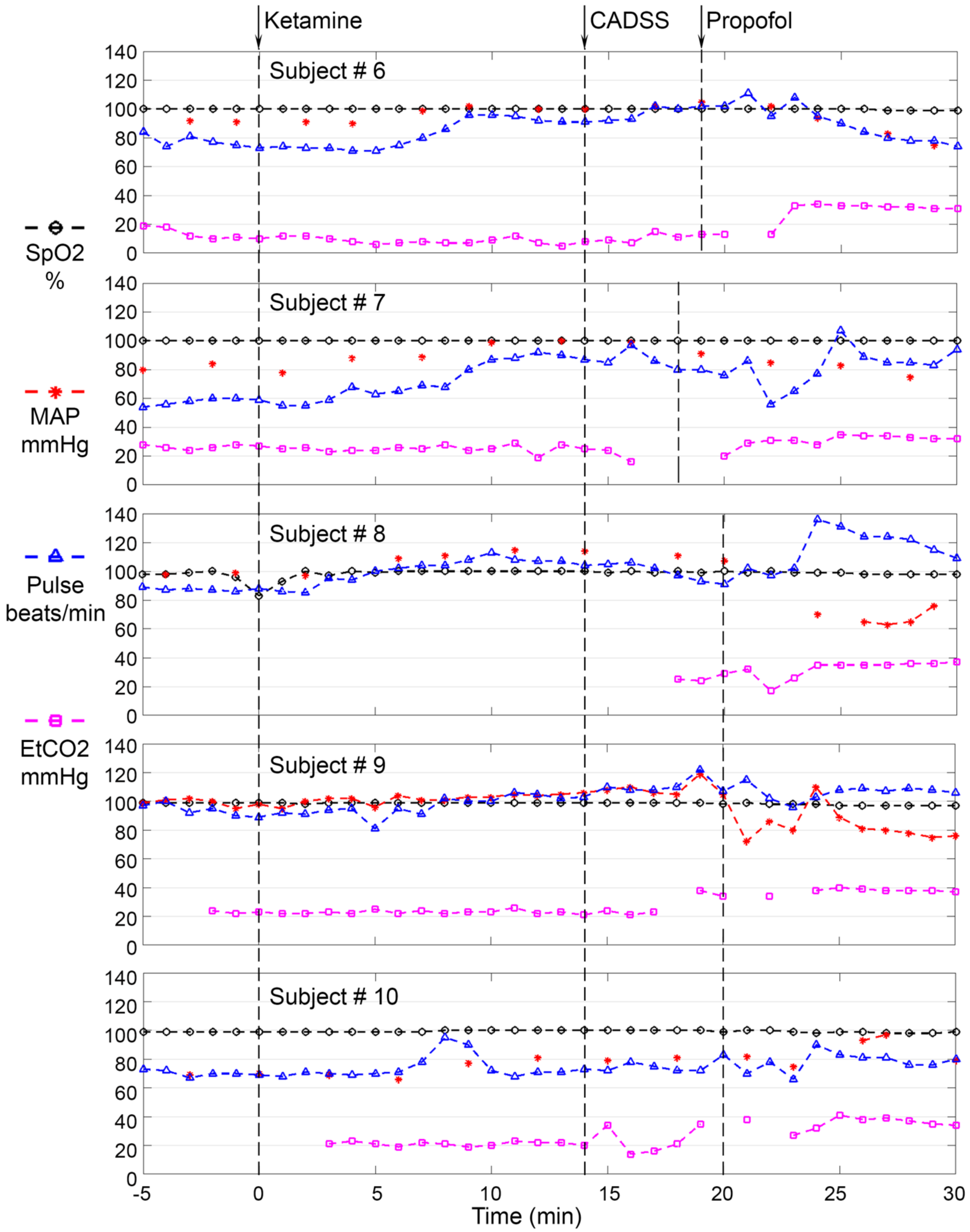

Supplementary Figure 8. Oxygen saturation (SpO2), mean arterial pressure (MAP), pulse, and end-tidal CO2 (EtCO2) for each period of the study. Information is not available for subjects #1-5. Source data are provided as a Source Data file Supplementary Figure 8.
