# Supplementary material for: Characterizing brain dynamics during ketamine-induced dissociation and subsequent interactions with propofol using human intracranial neurophysiology
Source: Nat Commun. 2023 Mar 29;14:1748. doi: 10.1038/s41467-023-37463-3 (PMC10060225; doi:10.1038/s41467-023-37463-3)
Supplement: Supplementary file 3 — Description of Additional Supplementary Files [file 41467_2023_37463_MOESM3_ESM.pdf]

File name: Supplementary Movie 1

Description: 3-Dimensional view of intracranial electrodes from all subjects on Colin 27 brain template
