# Supplementary material for: Characterizing brain dynamics during ketamine-induced dissociation and subsequent interactions with propofol using human intracranial neurophysiology
Source: Nat Commun. 2023 Mar 29;14:1748. doi: 10.1038/s41467-023-37463-3 (PMC10060225; doi:10.1038/s41467-023-37463-3)
Supplement: Supplementary file 5 — Reporting Summary [file 41467_2023_37463_MOESM5_ESM.pdf]

nature portfolio

Corresponding author(s): Patrick Purdon

Last updated by author(s): 02/21/2023

Reporting Summary

Nature Portfolio wishes to improve the reproducibility of the work that we publish. This form provides structure for consistency and transparency in reporting. For further information on Nature Portfolio policies, see our Editorial Policies and the Editorial Policy Checklist.

Statistics

For all statistical analyses, confirm that the following items are present in the figure legend, table legend, main text, or Methods section.

n/a	Confirmed	
☐	☒	The exact sample size (*n*) for each experimental group/condition, given as a discrete number and unit of measurement
☐	☒	A statement on whether measurements were taken from distinct samples or whether the same sample was measured repeatedly
☒	☐	The statistical test(s) used AND whether they are one- or two-sided<br>*Only common tests should be described solely by name; describe more complex techniques in the Methods section.*
☒	☐	A description of all covariates tested
☒	☐	A description of any assumptions or corrections, such as tests of normality and adjustment for multiple comparisons
☐	☒	A full description of the statistical parameters including central tendency (e.g. means) or other basic estimates (e.g. regression coefficient) AND variation (e.g. standard deviation) or associated estimates of uncertainty (e.g. confidence intervals)
☒	☐	For null hypothesis testing, the test statistic (e.g. *F*, *t*, *r*) with confidence intervals, effect sizes, degrees of freedom and *P* value noted<br>*Give P values as exact values whenever suitable.*
☒	☐	For Bayesian analysis, information on the choice of priors and Markov chain Monte Carlo settings
☒	☐	For hierarchical and complex designs, identification of the appropriate level for tests and full reporting of outcomes
☒	☐	Estimates of effect sizes (e.g. Cohen's *d*, Pearson's *r*), indicating how they were calculated

Our web collection on statistics for biologists contains articles on many of the points above.

Software and code

Policy information about availability of computer code

Data collection	Data were collected using the built-in software in Blackrock Cerebus system (Blackrock 416 Microsystems).
Data analysis	Data analysis was performed using custom analysis code in MATLAB (R2021a). Reconstruction of electrode locations were performed using the open-source, free software MMVT (https://github.com/pelednoam/mmvt) and Freesurfer (7.2). Simple line-art brain plots were produced using the open-source, free software Simple-Brain-Plot (https://github.com/dutchconnectomelab/Simple-Brain-Plot). Neural circuit plots were produced using Motifolio (license purchased).

For manuscripts utilizing custom algorithms or software that are central to the research but not yet described in published literature, software must be made available to editors and reviewers. We strongly encourage code deposition in a community repository (e.g. GitHub). See the Nature Portfolio guidelines for submitting code & software for further information.

Data

Policy information about availability of data

All manuscripts must include a data availability statement. This statement should provide the following information, where applicable:

- Accession codes, unique identifiers, or web links for publicly available datasets
- A description of any restrictions on data availability
- For clinical datasets or third party data, please ensure that the statement adheres to our policy

The data that support the findings of this study are available on request from the corresponding author. The raw data are not publicly available due to restrictions

relating to the per-participant imaging data currently containing information that could compromise the privacy of research participants. Source data are provided with this paper. The Desikan-Killiany-Tourville (DKT) 40 classifier atlas are available at http://www.mindboggle.info/data.html

Human research participants

Policy information about studies involving human research participants and Sex and Gender in Research.

Reporting on sex and gender	Ten patients (five male and five female) were recruited. Findings in this study apply to both gender. Both male and female were considered in study design. Gender information was obtained via patients' chart.
Population characteristics	Patients diagnosed with medication-refractory epilepsy participated in this study. These patients were implanted with intracranial depth electrodes to locate their seizure onset zone. Patients aged 22 to 59 years old were recruited.
Recruitment	Patients, age 18 to 70, were eligible for this study if they had already scheduled to undergo invasive clinical intracranial recording for the determination of epileptogenic origin. After a patient decided to undergo invasive monitoring, the treating epileptologist or surgeon would request permission of the patient to be contacted for this research study. If permission is granted by the patient to be contacted, he or she will be initially informed about the study. No self-selection bias or other biases exist.
Ethics oversight	Mass General Brigham Human Research Committee

Note that full information on the approval of the study protocol must also be provided in the manuscript.

Field-specific reporting

Please select the one below that is the best fit for your research. If you are not sure, read the appropriate sections before making your selection.

☒ Life sciences ☐ Behavioural & social sciences ☐ Ecological, evolutionary & environmental sciences

For a reference copy of the document with all sections, see nature.com/documents/nr-reporting-summary-flat.pdf

Life sciences study design

All studies must disclose on these points even when the disclosure is negative.

Sample size	No formal sample size calculation was performed as we did not know a priori the effect sizes of the changes in iEEG features that we would encounter. Instead, we referred to previous studies employing similar data to estimate the number of subjects to study. Ten patients constitute a substantial quantity of data for intracranial electrode recordings in humans, and many highly regarded, high-profile papers have studied fewer (n = 3) or similar (n = 13) number of patients [1-4].<br>[1] M J Kahana et al., Human theta oscillations exhibit task dependence during virtual maze navigation. Nature. 1999 Jun 24;399(6738):781-4. [NOTE: N = 3 subjects were studied]<br>[2] Rachel A Mak-McCully et al., Coordination of cortical and thalamic activity during non-REM sleep in humans. Nat Commun. 2017 May 25;8:15499. [NOTE: N = 3 subjects were studied]<br>[3] Martin Seeber et al., Subcortical electrophysiological activity is detectable with high-density EEG source imaging. Nat Commun. 2019 Feb 14;10(1):753. [NOTE: N = 3 subjects were studied]<br>[4] Hanna Hayat et al., Reduced neural feedback signaling despite robust neuron and gamma auditory responses during human sleep. Nat Neurosci. 2022 Jul;25(7):935-943. [NOTE: N = 13 subjects were studied]
Data exclusions	No subjects were excluded from the analysis; Individual recording channels were excluded from analysis if excessive noise was evident from visual inspection.
Replication	N/A (not a clinical trial; studies were performed on a single sample of relatively rare patients under going a surgical procedure)
Randomization	N/A (not a clinical trial; enrolled subjects required general anesthesia for their electrode explant procedure; baseline period prior to drug administration served as a within-subject control for the drug effect)
Blinding	N/A (not a clinical trial; enrolled subjects required general anesthesia for their electrode explant procedure and were informed that they would be receiving ketamine as part of the informed consent procedure; clinicians were also required to know that they were administering ketamine in order to manage the clinical effects of the drug)

Reporting for specific materials, systems and methods

We require information from authors about some types of materials, experimental systems and methods used in many studies. Here, indicate whether each material, system or method listed is relevant to your study. If you are not sure if a list item applies to your research, read the appropriate section before selecting a response.

Materials & experimental systems

n/a	Involved in the study
☒	☐ Antibodies
☒	☐ Eukaryotic cell lines
☒	☐ Palaeontology and archaeology
☒	☐ Animals and other organisms
☒	☐ Clinical data
☒	☐ Dual use research of concern

Methods

n/a	Involved in the study
☒	☐ ChIP-seq
☒	☐ Flow cytometry
☐	☒ MRI-based neuroimaging

Magnetic resonance imaging

Experimental design

Design type: Structural imaging

Design specifications: N/A (only structural imaging was performed, i.e., no fMRI)

Behavioral performance measures: N/A (only structural imaging was performed, i.e., no fMRI)

Acquisition

Imaging type(s): Structural

Field strength: Siemens Trio 3 Tesla

Sequence & imaging parameters: T1-weighted magnetization-prepared rapid gradient echo, 1.3-mm slice thickness, 1.3×1 mm in-plane resolution, TR/TE=2530/3.3 ms, 7° flip angle

Area of acquisition: Whole brain

Diffusion MRI: ☐ Used ☒ Not used

Preprocessing

Preprocessing software: Freesurfer (7.2)

Normalization: N/A (only structural imaging was performed, i.e., no fMRI)

Normalization template: N/A (only structural imaging was performed, i.e., no fMRI)

Noise and artifact removal: N/A (only structural imaging was performed, i.e., no fMRI)

Volume censoring: N/A (only structural imaging was performed, i.e., no fMRI)

Statistical modeling & inference

Model type and settings: N/A (only structural imaging was performed, i.e., no fMRI)

Effect(s) tested: N/A (only structural imaging was performed, i.e., no fMRI)

Specify type of analysis: ☒ Whole brain ☐ ROI-based ☐ Both

Statistic type for inference (See Eklund et al. 2016): N/A (only structural imaging was performed, i.e., no fMRI)

Correction: N/A (only structural imaging was performed, i.e., no fMRI)

Models & analysis

n/a	Involved in the study
☒	☐ Functional and/or effective connectivity
☒	☐ Graph analysis
☒	☐ Multivariate modeling or predictive analysis
